# Can the Revolution in mRNA-Based Vaccine Technologies Solve the Intractable Health Issues of Current Ruminant Production Systems?

**DOI:** 10.3390/vaccines12020152

**Published:** 2024-01-31

**Authors:** Timothy J. Mahony, Tatiana E. Briody, Sheila C. Ommeh

**Affiliations:** Centre for Animal Science, Queensland Alliance for Agriculture and Food Innovation, The University of Queensland, Brisbane, QLD 4072, Australia; t.briody@uq.edu.au (T.E.B.); s.ommeh@uq.edu.au (S.C.O.)

**Keywords:** animal health, infectious disease, vaccination, mRNA vaccine, lipid nanoparticle, veterinary health, lumpy skin disease virus, rift valley fever virus, peste des petits virus, east coast fever, *Theileria parva*, bovine respiratory disease, barber’s pole worn, *Haemonchus contortus*

## Abstract

To achieve the World Health Organization’s global Sustainable Development Goals, increased production of high-quality protein for human consumption is required while minimizing, ideally reducing, environmental impacts. One way to achieve these goals is to address losses within current livestock production systems. Infectious diseases are key limiters of edible protein production, affecting both quantity and quality. In addition, some of these diseases are zoonotic threats and potential contributors to the emergence of antimicrobial resistance. Vaccination has proven to be highly successful in controlling and even eliminating several livestock diseases of economic importance. However, many livestock diseases, both existing and emerging, have proven to be recalcitrant targets for conventional vaccination technologies. The threat posed by the COVID-19 pandemic resulted in unprecedented global investment in vaccine technologies to accelerate the development of safe and efficacious vaccines. While several vaccination platforms emerged as front runners to meet this challenge, the clear winner is mRNA-based vaccination. The challenge now is for livestock industries and relevant stakeholders to harness these rapid advances in vaccination to address key diseases affecting livestock production. This review examines the key features of mRNA vaccines, as this technology has the potential to control infectious diseases of importance to livestock production that have proven otherwise difficult to control using conventional approaches. This review focuses on the challenging diseases of ruminants due to their importance in global protein production. Overall, the current literature suggests that, while mRNA vaccines have the potential to address challenges in veterinary medicine, further developments are likely to be required for this promise to be realized for ruminant and other livestock species.

## 1. Introduction

### 1.1. Sustainable Development Goals

The debate continues about the number at which the global human population will peak towards the end of the 21st century, with estimates ranging from 8.7 to 10.7 billion [1]. Regardless, there is a clear need for the increased production of high-quality protein for human consumption to achieve the Sustainable Development Goals (SDGs) as defined by the United Nations. This increase in edible protein productivity would make an important contribution towards meeting the three SDGs that concern poverty (SDG 1), hunger (SDG 2), and good health (SDG 3). At the same time, other SDGs that address responsible production (SDG 12), climate action (SDG 13), and the use of land and water resources (SDG 14 and 15) could be seen to act in opposition to SDG-1 to 3. Recently, there has been an increase in the consumption of protein in countries with emerging economies, such as those in Asia and South America [2,3]. Historically, the response to demands for increased quantities of an agricultural commodity of interest has been to expand the production footprint. It is now readily apparent that this approach is no longer sustainable, and new innovations are required to meet these challenges [4]. One way to address these potentially conflicting SDGs is to improve the efficiency of current animal-based farming by addressing the losses in production due to diseases.

Pathogens and pests that cause disease are major limiters of production in both terrestrial and aquatic livestock systems [5,6]. The use of safe and efficacious vaccines has underpinned improvements in both human and animal health [7]. An additional benefit of safe and effective vaccines for livestock diseases is the prevention of antimicrobial-based treatments in food-producing animals. The global emergence of antimicrobial resistance in microbes of importance to human medicine has brought into focus the use of these drugs in food-producing animals [8]. This is reflected in the increasing pressure on livestock systems to implement antimicrobial stewardship policies to limit the spread of drug-resistant microbes in the food supply chain [9].

This review aims to describe the key features of mRNA vaccines and how the further optimization of this rapidly maturing technology could enable the improved control of important infectious livestock diseases. Although mRNA technology is likely to be highly suited for use in most, if not all, livestock species, this review focuses on the diseases of ruminants. Ruminants have an unmatched capacity to convert low-quality forage into high-quality protein, suggesting that they will remain an important component of global food production for the foreseeable future [10]. The challenges of adapting mRNA vaccination as it continues to rapidly mature to address the important diseases of ruminants, that, if adequately controlled, would contribute to achieving the SDGs, are also discussed.

### 1.2. Veterinary Vaccine Development

Historically, the development of veterinary vaccines has followed a rather simplistic paradigm. This paradigm could be described as investigate, isolate, propagate, inactivate/attenuate, vaccinate, and evaluate. Following the emergence or identification of an infectious disease affecting livestock, an investigation will be undertaken to identify the type of pathogen involved and to determine if it can be propagated in the laboratory. If the pathogen is amenable to these processes, in-depth laboratory studies are likely to follow to further characterize it. If the need is deemed to be high enough, the development of a vaccine based on the pathogen isolate may follow along one of two pathways, the first of which is the large-scale culture of the pathogen followed by the inactivation and formulation into a prototype vaccine. A disadvantage of this approach is the inactivated pathogens are poorly immunogenic, and therefore require formulation with adjuvants to ensure the stimulation of the host’s immune system [11].

The second commonly used pathway is pathogen attenuation. This can be achieved by the serial passage of the pathogen under conditions that produce a derivative which has lost the capacity to cause disease in the target host. Referred to as modified live vaccines (MLVs), these types of vaccines are highly effective, as they mimic the natural infection cycle, thus stimulating similar immune responses to that of the native pathogen without the negative effects of the disease. Many highly effective veterinary vaccines have been produced using these approaches [12]. Equally, many infectious diseases have proven to be resistant, or not amenable, to these classical developmental pipelines.

Subunit vaccines are another approach that has received attention for the development of veterinary vaccines [13]. A subunit vaccine consists of one or more components of the pathogen of interest that is known to confer protection from disease when recognized by the host immune system. For diseases associated with viruses, the viral proteins that harbor the neutralizing epitopes have been widely studied in the development of this class of vaccine [14]. While the subunit can be purified from a pathogen, it is likely that the use of recombinant DNA technology will be a more cost-effective production system. The subunit antigen of interest will likely guide the choice of the expression system. A key advantage of subunit vaccines is that they are considered safe, as there are no risks of reversion to virulence [12]. Disadvantages include the cost of production and the need to formulate the subunit with adjuvants to improve the immunogenicity.

Other vaccine technologies have been developed to address some of the deficiencies, such as recombinant vectored and DNA vaccines [14]. Few of these have progressed to commercialization and industry adoption for a variety of issues, including the poor efficacy and the high cost of production. However, as discussed later in this review with specific examples, current vaccine technologies, such as MLVs and subunit vaccines for ruminants, have the potential to inform the design of mRNA vaccines for these species.

### 1.3. Emergence of mRNA Vaccination

The COVID-19 pandemic resulted in unprecedented investment to accelerate the development, evaluation, commercialization, and deployment of an effective vaccine [15]. The clear winner of this process was mRNA vaccines. Despite the widely held perception, mRNA vaccines were not developed in response to the pandemic; rather, it was an existing platform that could be readily adapted to meet the need for a safe and efficacious vaccine against the rapid emergence of severe acute respiratory syndrome coronavirus 2 (SARS-CoV-2). Malone et al. [16] were the first to report the successful delivery and translation of an in vitro-produced mRNA into the cytoplasm of mammalian, amphibian, and insect cells using liposomes. The study also identified the critical roles of the various structural elements of an mRNA that can increase the translation efficiency of an encoded polyprotein, which will be examined further below [16]. Soon thereafter, Wolff et al. [17] reported that, even in the absence of a carrier technology, mRNA alone injected into the muscles of mice was taken up by the cells, resulting in the translation of the encoded polypeptides. Whilst this study demonstrated the feasibility of direct in vivo mRNA delivery, it is now widely accepted that the use of a carrier technology is the preferred approach for the optimal efficiency of the delivery, stability, and translation of mRNA.

While the early focus of mRNA applications was for therapeutic use, it was soon realized that the approach could also be a promising new vaccine technology. Initially, Martinon et al. [18] reported the liposome-mediated transfer of mRNA encoding the influenza nucleoprotein was able to induce cytotoxic lymphocyte responses in mice that were indistinguishable from those elicited by infection with the virus. The study also demonstrated that an mRNA-based vaccine could elicit peptide-specific cytotoxic immune responses in mice with different major histocompatibility complex (MHC) haplotypes [18]. It was later reported that 71% of mice (*n* = 7) immunized with 50 µg of an mRNA encoding for the human carcinoembryonic antigen developed specific antibodies to the expressed immunogen [19]. Following these early studies, the first assessment of mRNA encoding tumour antigens was the injection of naked mRNA adjuvanted with granulocyte macrophage colony-stimulating factor in phase I/II clinical trials reported by Weide et al. [20]. While the formulation was deemed to be safe, antitumour responses were not detected in all participants [20]. Although many of these landmark studies were focused on immunization with cancer antigens, collectively they demonstrated the potential for in vitro-transcribed (IVT) mRNA to underpin development of an effective and flexible immunization technology. While the key technical aspects of mRNA vaccination will be discussed here, it is beyond the scope of this review to present a comprehensive developmental timeline analysis of mRNA-based vaccines. The reader is instead directed to several detailed review articles on this subject [21,22,23].

### 1.4. Comparative Cost of mRNA Vaccines

The cost of a veterinary vaccine can be a be a key driver of end-user adoption, particular in developing countries [24]. As there are currently no mRNA vaccines registered for use in ruminants or other livestock species, Table 1 compares the estimated cost of dose production for mRNA vaccines to selected vaccination platforms for use in humans. This comparison suggests the quantity of active components (e.g., mRNA) and the scale of production are important drivers of the end cost of a dose (Table 1). The impact of the quantity of active components required in a dose is clearly demonstrated by the low costs associated with self-amplifying RNA (Table 1). Self-amplifying RNAs are similar to mRNA, but have the capacity for the RNA-directed in vivo synthesis of the mRNA-encoding antigen through *cis*- or *trans*-acting viral components [25]. The comparisons suggest that the cost of producing a dose of an mRNA vaccine compares favourably to those technologies currently used to produce veterinary vaccines (Table 1). The cost of producing an mRNA dose for use in ruminants and humans is likely to be similar, as approximately 70% of the cost of production is the raw ingredients that will be the same across both applications [26]. The cost estimates for the bacterial, viral, and subunit vaccines were based on production in a developing country (Table 1). The cost of producing these same vaccines in a developed country would likely increase due to the higher labour- and infrastructure-associated costs [27].

## 2. Design and Delivery of mRNA Vaccines

### 2.1. mRNA Vaccine Components

There are two key components of an mRNA vaccine. The first component is the mRNA that encodes an antigen(s) of the pathogen of interest. The role of the mRNA is to direct the synthesis of the antigen to enable processing by the immune system to direct the development of specific and protective immunological responses. The second component is an appropriate delivery system, as typically RNA molecules have limited stability and are not able to passively transit through the outer membrane of mammalian cells with great efficiency.

**Figure 1:**
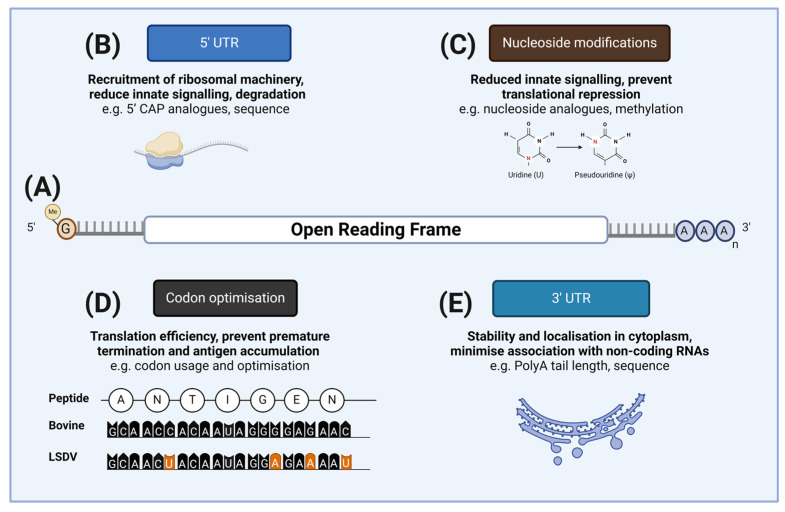
Schematic representation of the essential structural elements of an mRNA used in vaccine formulation. Selected modifications that can be used to optimize the in vivo performance of an mRNA are illustrated. Examples and roles of these elements are also shown. (**A**) The linear arrangement of the essential components of an mRNA vaccine. (**B**) 5′-Untranslated region (5′-UTR). (**C**) Nucleoside modifications. (**D**) Codon optimization, including an example of the different codon usages of bovines and lumpy skin disease virus (LSDV) for a theoretical peptide antigen. (**E**) 3′-Untranslated region (3′-UTR). Image produced with BioRender™.

The basic design of the mRNA component is largely based on the accepted structure of eukaryotic mRNAs. Simplistically, there are five structural components of the mRNA that forms the active component of an mRNA vaccine: the 5′ cap structure, the 5′-untranslated region (5′-UTR), the open reading frame (ORF) that encodes the antigen(s) of interest, the 3-untranslated region (3′-UTR), and the polyadenylated tail (polyA) (Figure 1). These components contribute to the stability and translation efficiency of the mRNA of interest, and may have an impact on antigen production, and therefore the elicited immune responses. There are several excellent reviews that comprehensively address these components [30,31] and, as such, they will not be reviewed in detail here. Rather, the key elements of each are briefly summarized below to frame the following discussion on how this technology could be applied to the key veterinary diseases discussed in this review.

All eukaryotic mRNAs have a 5′ cap structure that is added to the nascent RNA soon after transcription begins in the nucleus of cells [32]. Specifically, there are two structural components to the mRNA cap. The first, cap 0, is an N7-methylated guanosine linked to the first nucleotide (N0) of the mRNA via a reverse 5′ to 5′ triphosphate linkage, added by a multienzyme pathway. The second component, cap 1, is the 2′O methylation of the second mRNA nucleotide (N + 1). The cap 0 is involved in the nuclear processing of mRNA, including splicing, polyadenylation, and nuclear export. In the context of mRNA vaccines, the cap 0 plays crucial roles in preventing the degradation by 5′ to 3′ nucleases, the recruitment of the proteinaceous translation components, and the formation of mRNA tertiary structures that affect translation efficiencies, and therefore antigen production. The cap 1 is also of importance with respect to mRNA vaccines, as it prevents innate immune signalling by facilitating the recognition of the mRNA as a “self”. While it might be considered beneficial for a vaccine to have an inherent capacity to stimulate immune responses, it is certainly not the case for mRNA vaccines. These pathways are responsible for the sensing of foreign RNA molecules and are focused on the detection of uncapped pathogen RNAs, particularly viral RNAs. One consequence of activating these pathways is the induction of an interferon response that can result in the inhibition of translation, and therefore reduced antigen production [33].

The 5′-UTR sequences of eukaryotic mRNAs are crucial in the recruitment of the proteinaceous components of the ribosome and the subsequent initiation of protein translation [34]. The recruitment of these factors is dependent on the secondary structures formed by the 5′-UTR via Watson–Crick–Franklin and noncanonical base-pairing. Indeed, the overall tertiary structure of not just the 5′-UTR but the complete mRNA can impact the translation efficacy, and therefore the antigen production, in the context of mRNA vaccines. This is an aspect of mRNA vaccine design that will be discussed further below.

A critical component of an mRNA vaccine is the ORF that encodes the antigen of the target pathogen. In many instances, pre-existing subunit vaccines could be readily “converted” to mRNA vaccines. The development of mRNA vaccines against veterinary viruses could be highly amenable to this approach, as, for many of these viruses of economic importance, the viral components involved in eliciting protective immune responses are known or could be extrapolated from the viruses of other species. This was exemplified during the recent global pandemic by the rapid design of mRNA vaccines targeting SARS-CoV-2 [35]. Once the virus was identified as a coronavirus, vaccine development efforts were able to focus on the spike protein based on the prior knowledge of the crucial role that this antigen played in inducing protective immunological responses against viruses in this family. Once identified, the antigen ORF may undergo an optimization process to improve the translation efficiency, and therefore improve antigen production in vivo. A common feature in the heterologous expression of antigens is to optimize the codon usage patterns of pathogen genes to be more similar to that of the expression system used or, in the case of mRNA vaccination, the host to be immunized (Figure 1). There is considerable redundancy in the trinucleotide codons that encode natural amino acids; thus, the patterns of codon usage by the organisms, including pathogens, are not random, a phenomenon commonly referred to codon usage bias [36]. A common starting point in the development of the mRNA vaccine ORF is to alter the native codon usage bias to reflect the codon usage patterns of the host that is to be vaccinated. The complexity of this aspect of mRNA design and optimization was illustrated by a recent study that applied quantum computing processes to codon optimization to improve antigen expression [37].

The codon bias of an ORF can be used to modulate translation in several ways. It might be expected that the complete optimization of the ORF to match the codon composition with highly expressed tRNAs within a target tissue would improve the translation efficacy, and therefore increase antigen production. However, if translation occurs too rapidly, there is an increased risk of protein misfolding that may be detrimental to antigen production and/or lead to antigen accumulation within the cell [38]. In contrast, reduced translation efficiency through the inclusion of infrequently used or rarely used codons can result in the premature termination of translation, which will also negatively affect antigen production [39,40]. It is therefore evident that not only is codon usage an important step in the optimization of an antigen ORF, but also codon distribution throughout the ORF. Consequently, a common strategy for codon optimization is to adopt the usage patterns of the genes that are known to be efficiently transcribed in the host or even the tissue or cell type that the mRNA will be delivered to.

The 3′-UTR plays key roles in mRNA localization, half-life/stability, and translation efficiency. While it is beyond the scope of this review, the interaction of the 3′-UTR with small noncoding RNA molecules, particularly microRNA, are thought to underpin the post-transcriptional regulation of the complex gene expression associated with higher eukaryotes [41]. Thus, the 3′-UTR elements used in any mRNA must be carefully considered to minimize the risk of unintended interactions with microRNAs that might adversely affect antigen translation.

The final domain of canonical mRNAs is the polyadenylated (polyA) tail, a defining characteristic of eukaryotic mRNA molecules. The mRNA polyA tail plays critical roles in modulating the translation efficiency and stability of the molecule in the cytoplasm [42].

### 2.2. mRNA Vaccine Construction and Synthesis

Once the primary design of the mRNA molecule is finalized, it will typically be constructed as an insert in a bacterial plasmid to facilitate construction and validation by sequencing. How the plasmid is constructed will vary depending on the laboratory. As an example, a laboratory may have an existing reporter plasmid backbone with all the required regulatory elements described above that has been constructed and used with their disease model and/or relevant host. The reporter ORF can then be replaced with the ORF encoding the antigen of interest. An additional element may need to be added to the construct to facilitate the production of transcripts, such as the bacteriophage T7 or SP6 promoter sequence immediately proximal to the selected 5′-UTR element. While it is possible to polyadenylate RNA molecules post-transcriptionally, it can be difficult to control the extent of polyadenylation, and therefore predict how variations might impact the translation efficiency and/or mRNA half-life [42]. Whereas, if the optimal sequence composition of the polyA for the mRNA of interest is known or has been empirically determined, the construct template can be developed to replicate this. The final element that is required for the plasmid construct is the incorporation of a unique restriction endonuclease site to facilitate the linearization of the plasmid. While transcripts can be generated using circular templates, the highly processive capacities of bacteriophage RNA polymerases can result in highly variable transcription products. Clearly, from a vaccine formulation and antigen translation perspective, uniformity in the mRNA product is critical.

The current leading technology for mRNA vaccine synthesis is IVT. From a veterinary vaccine regulatory perspective, this is an ideal approach, as all the components used in the manufacture of the mRNA can be accurately defined to manage risks, such as those posed by adventitious agents [43]. The recent production of T7 RNA polymerase for IVT without the use of any animal products reinforces this advantage [44]. Similarly, IVT also enables the optimization of reaction components if required to improve the yield and/or composition of the mRNA of interest. IVT can readily facilitate the substitution and/or optimization of reaction components if required. An important example of this is the use of nucleotide analogues. As previously described, the presence of the CAP structure and the 5′ terminus of the mRNA plays a crucial role in the self-recognition of eukaryotic mRNA molecules. Similarly, the use of natural nucleotides during the IVT production of an mRNA can result in the stimulation of innate immune responses when these molecules are introduced into host cells. This innate immune signalling is mediated by multiple components within the cell. While some innate signalling is considered essential with respect to mobilizing adaptive immune responses, overstimulation can lead to reactogenicity that can be counterproductive with respect to the therapeutic uses of mRNA. The seminal work of Kariko et al. [45] demonstrated that the post-transcriptional modifications of the nucleotides in mammalian mRNA molecules, such as methylation, significantly reduced innate immune signalling mediated by RNA sensing by Toll-like receptors 3, 7, and 8. The substitution of unmodified nucleotides with naturally occurring methylated nucleotides or the uridine derivative pseudouridine [Ψ] significantly reduced innate immune signalling, with reduced cytokine and activation marker expression detected [45]. Of those evaluated, m5U, s2U, and pseudouridine were the most effective in preventing and/or dampening the mRNA-mediated stimulation of innate immune signalling and translational repression [46,47]. A key outcome of using modified nucleotides, particularly pseudouridine, was the lessened expression of 2′-5′-oligoadenylate synthetase and RNase L, which are expressed as a result of interferon signalling, the result of which was the increased translation and half-life for the modified mRNA [48]. The importance of this body of work is further illustrated by the widely used COVID-19 mRNA vaccines utilizing N1-methylpseudouridine (m1Ψ), which have a higher reported efficacy compared to a third vaccine that does not [49,50].

Once the sequence of the plasmid construct is confirmed, the plasmid is linearized as close as is practicable to the 3′ terminus of the polyA sequence by digestion with a type IIS restriction endonuclease. The type IIS restriction enzymes cleave double-stranded DNA downstream of their recognition site in a sequencing-independent manner and, as such, the integrity of the sequence of interest, in this case the polyA motif, is maintained. The linearized plasmid is then utilized as a template for in vitro transcription using the bacteriophage RNA polymerase (T7 or SP6) that matches the promoter sequence included in the plasmid construct. The RNA CAP structure can then be added to the mRNA using one of various approaches, including simultaneous transcription and capping, as reviewed by Muttach et al. [51]. Once the transcription reaction is complete, the plasmid template can be removed by DNase treatment and the mRNA can be recovered using conventional RNA extraction methods. The mRNA can then be purified from the other components of the reaction. This is a critical step, as many of the in vitro transcription reaction components are likely to act as triggers for innate immune signalling.

### 2.3. mRNA Stabilization and Cellular Delivery

Following production and purification, the mRNA is then ready for vaccine dose formulation. As mammalian cells are not permissive to passive mRNA uptake, various technology platforms have been evaluated to deliver the mRNA to the cytoplasm for the translation of the encoded antigen. The inability of mammalian cells to directly take up RNA molecules was clearly demonstrated in a study investigating the development of an mRNA for rabies virus in a phase I clinical trial [52]. The study compared the delivery of an mRNA encoding the rabies virus viral fusion protein either intradermally or intramuscularly via two methods. The first delivery method evaluated utilized conventional needle injection, with one of forty-two participants generating detectable immune responses. In contrast, when the mRNA was delivered using pressurized needle-free devices, intradermally (*n* = 45) or intramuscularly (*n* = 13), strong virus-neutralizing antibody responses were detected in 71% and 46% of participants, respectively [52]. The results of this study clearly demonstrate the need for an effective strategy to facilitate the delivery of mRNA into cells to facilitate antigen translation. Currently, the leading technology for the delivery of mRNA vaccine molecules is ionizable LNPs. The utility of LNP delivery systems will be demonstrated through practical examples throughout this review.

When considered together, there are clearly a multitude of factors across the various mRNA domains that must be considered when developing a prototype mRNA vaccine. Currently, the typical starting point is to select a well-characterized transcript that is known to be efficiently translated, leading to the production of high levels of the encoded polypeptide in the tissue that the vaccine is likely to be administered to. The starting mRNA construct can then be designed to include the key structural motifs described above, replacing the native ORF with the ORF encoding the antigen of interest. There are several emerging strategies discussed in this review that aim to improve the efficiency of the mRNA design processes.

Various technologies have been utilized to enable the stabilization and efficient delivery of mRNA molecules for the purpose of vaccination. The structural and physiological properties of the outer membrane of mammalian cells, such as the hydrophilicity and negative charge, make it largely refractile to the passive uptake of mRNA molecules. In addition, the presence of nucleases, including RNases, in the extracellular environments of tissues can lead to the degradation of mRNA prior to cell entry. A selected range of technologies used to address these issues are briefly described here. More detailed descriptions can be found in recent reviews specific to this aspect of mRNA vaccine development [21,53,54,55].

One of the earliest mRNA delivery systems was peptide carriers, known as protamines. Protamines are composed predominantly of the positively charged amino acid arginine that associates with DNA in male gametes [56]. As mRNA molecules are negatively charged, they can form complexes with the protamine carriers based on electrostatic interactions, thus providing some protection from RNases. It has been suggested that these complexes are potentially self-adjuvating due to the stimulation of the innate immune system through Toll-like receptor 7 sensing. Fotin-Mleczek et al. [57] report that an mRNA encoding the model antigen OVA was able to elicit balanced antigen-specific antibody and cell-mediated responses in this way using a murine OVA–tumour model. The capacity of the protamine mRNA to simulate strong innate responses has been hypothesized to result from the complexes mimicking the nucleocapsids of RNA viruses [58].

Various polymers have also been used to protect and enable the bioavailability of mRNA vaccines. Cationic polymers, such as polyethylenimine (PEI), polyamidoamine, and polysaccharides, have been investigated for their improved mRNA stability and delivery. Polyethyleneimine can be formulated with mRNA by the direct mixing of solutions. Li et al. [59] reported the use of cyclodextrin–PEI (CP-2k) conjugates, having been evaluated for the targeting of human immunodeficiency virus in a mouse model. The study compared the persistence of mRNA, mRNA–PEI, and mRNA–CP-2k following intranasal installation [59]. The results suggested the prolonged retention of the mRNA–CP-2k at the nasal mucosa and the increased mRNA uptake by the nasal-associated lymphoid tissue [59]. The study also demonstrated that the mRNA–CP-2k formulation was able to prevent experimental-induced toxicity due to the nonspecific uptake of exogenous lipopolysaccharide, whereas the mRNA–PEI did not. This difference was reported to result from the reversible opening of tight cell junctions to promote paracellular delivery while not disrupting the integrity of the nasal epithelia [59]. Importantly, the mRNA–CP-2k formulation also induced significantly stronger serum IgG responses compared to the other treatments. Of particular note in this study was that the mRNA–CP-2k and the PEI–mRNA treatments induced similar secretory IgA responses at the nasal mucosa. However, the mRNA–CP-2k group had significantly higher secretory IgA in vaginal washes compared to the PEI–mRNA-treated group. The mRNA–CP-2k-immunized mice also had significantly higher cellular-mediated responses, with higher cytotoxic T-cell activity, higher numbers of CD8+ secreting INF-γ, and CD4+ T-cells secreting IL-4 [59].

Further investigations suggest that mice immunized with mRNA–CP-2k elicited balanced Th1/Th2/T17 immune responses based on in vivo and in vitro cytokine analyses [60]. Importantly, these responses included detectable type I interferon, suggesting this formulation induced an innate immune response that did not hinder the subsequent development of robust adaptive immune responses. Immunization with mRNA alone induced strong type I interferon responses that may have resulted in the reduced translation and skewing of the immune response towards an inflammatory response, potentially hindering the development of adaptive immune responses [59]. There are several outcomes of this study that are important with respect to veterinary vaccine development. The study confirmed the immunological linkage between disparate mucosal sites, with the formulation inducing a significantly higher production of vaginal IgA following intranasal administration. This finding is important for the application of intranasal vaccination to address reproductive diseases, where injection-based approaches have proven unsuccessful in eliciting mucosal responses. While not examined in the study of Li et al. [59], it would also be of interest to determine if, when using this approach, mucosal responses were also elicited in the gastrointestinal tract, as there are multiple livestock diseases of economic importance that involve the gut lumen.

To date, the most widely applied delivery technology for mRNA-based vaccination has been lipid nanoparticles (LNPs). The commonly used elements of LNPs are either a cationic or ionizable lipid material, a zwitterionic lipid similar to that found in cell membranes, and a stabilizer of the lipid bilayer, such as cholesterol and polyethylene glycol to improve LNP dispersal and to limit the nonspecific protein adherence to the surface of the LNPs. More detailed descriptions of the key properties of the LNPs and their development for uses in biological systems, including the effective delivery of mRNA, are described by Blanco et al. [61].

As LNPs have been widely used in mRNA vaccination studies, there has been considerable interest in the mechanisms that underpin their capacity to enable the effective delivery of mRNA therapeutics and vaccines. The working hypothesis is that the LNPs attach to the cellular membrane via electrostatic interactions, resulting in the subsequent fusion of the lipid components. The initiation of cell entry is thought to be clathrin-dependent endocytosis and subsequent micropinocytosis-mediated endocytosis. After entry, the LNPs are trafficked from the early endosome to the late endosome, and then to the lysosome, where their mRNA cargo can be degraded. The mechanisms that underpin mRNA release to the cytoplasm to enable antigen translation are yet to be fully elucidated. A commonly held hypothesis for the observed functional delivery of mRNA, referred to as the proton sponge effect, is the disruption of the lysosomal membrane resulting from the acidification (a pH of 6.5 to a pH < 6) of the endosome lumen due to ATP-driven enzymic activity. The perturbation of the endosomal membrane allows a low percentage (<2%) of the LNPs to evade destruction, leading to the escape of the mRNA cargo [55]. Other factors may also influence the efficiency of the functional mRNA delivery. As an example, Patel et al. [62] demonstrated that the manipulation of mTORC1 signalling could inhibit or enhance the LNP-mediated delivery of mRNA.

### 2.4. Mechanisms of mRNA Endosomal Escape

To investigate the underpinning mechanisms of mRNA release from endosomes, Paramasivam et al. [63] compared the endosomal escape capacities of LNPs synthesized with six different cationic lipids. Using a reporter mRNA encoding GFP, the study demonstrated considerable variability in the efficiencies of the cellular uptake and subsequent translation of GFP. While there did appear to be a direct correlation between LNP–mRNA uptake and GFP expression, this was not consistent for all LNPs. The L606 LNP had the highest expression of the EGF reporter. However, the uptake of the L606 LNP–mRNA was significantly lower compared to the MC3 and ACU5 LNP formulations [63]. This suggests that the efficiency of the uptake is not the sole indicator for mRNA translation efficiency. The study also evaluated the lysosomal trafficking of the LNP formulations to address the hypothesis that the optimal LNP should deliver the mRNA to the compartment of the organelle, where it can be released for subsequent translation or degraded with minimal impacts on the function of these pathways. Using phenotypic protein markers for early/recycling endosome (Rab11), different types of early endosomes (EEA1+ and APPL1+), and late endosomes (LAMP1), quantitative differences were observed in the proportions of the LNP–mRNA formulations accumulating in these compartments. Significantly higher LNP accumulation was observed in both types of early endosomes positive for the EEA1 or APPL1 markers. For the more efficient LNPs described above (L608, MC3, and ACU5), a higher accumulation was observed in the early/recycling endosomes (Rab11+) and early endosomes (EEA1+ and APPL1+) compared to the low-efficiency LNPs. In contrast, there was minimal accumulation of these LNPs in the late endosomes (LAMP1+), which indicated that this compartment was a minor contributor to efficient mRNA delivery.

The study determined that the arresting of endosome maturation through acidification was linked to the quantity of accumulated LNP–mRNAs, with a higher accumulation linked to a more severe inhibition of acidification. Using an analysis of low-density lipoprotein (LDL) recycling via the lysosome as a model, it was also demonstrated that, for both the lower and higher levels of LNP–mRNA accumulation, cargo (LDL) degradation was similarly inhibited [63]. The study subsequently demonstrated that the impairment of acidification and cargo processing makes endosomes unsuitable for mRNA escape and cytoplasmic delivery [63]. To resolve the question of which substructures of endosomes are the major contributors to mRNA escape, and therefore mRNA vaccine efficiency, the authors utilized multicolour single-molecule localization microscopy (SMLM). On the basis that endosomes with the phenotypic markers Rab11+, APPL1+, and EEA1+ perform cargo transport and recycling to the plasma membrane using tubular structures, the authors evaluated if these could provide a viable pathway for mRNA escape to the cytosol. Using SMLM, the authors were able to resolve single LNPs, estimating between 5 and 25 mRNA copies per nanoparticle, and single mRNA molecules in cells. Fluorescently labelled transferrin and epidermal growth factor were used as markers of early and late endosomes, respectively. The results indicated that the LNPs were mostly associated with transferrin, suggestive of their localization in early-recycling endosome compartments [63].

The study also identified the colocalization of labelled mRNAs with the labelled transferrin of endosomal tubules for the four LNP formulations using SMLM. The pattern of mRNA fluorescence was considered consistent with the clustering to individual molecules. The single-molecule signals were closely associated with transferrin signals, suggesting the former were mRNAs released from the LNPs that were undergoing endosomal escape [63]. The study also observed linearly arranged mRNA fluorescent signals along the tubules that extended into the cytoplasm, which the authors interpreted as the actual moment of mRNA escape [63]. The authors concluded that the results of their study support a new mechanism for the critical step in mRNA vaccination, the mRNA endosomal escape to the cytoplasm to enable translation.

The results of this study clearly underscore the need to elucidate the mechanisms that underpin the capacities of different types of mRNA delivery technologies. Understanding these mechanisms can inform the further optimization of delivery to improve mRNA release from the endosomes. The flow-on impact of this could be to further reduce any deleterious effects, such as toxicity or the overstimulation of innate immune pathways that skew the immune response to inflammation rather than the desired balanced of adaptive immune responses. It is likely that the next leap in mRNA vaccine efficacy will come from the optimization of endosomal escape by mRNA, perhaps with the application of artificial intelligence and machine learning to fully understand the complex molecular interactions involved in endosomal trafficking and mRNA escape to the cytoplasm. Such an approach may identify commonalities and bottlenecks between different delivery technologies to enable the selection of the appropriate LNP strategy for the application of interest, including those in veterinary medicine.

## 3. Immune Responses to mRNA Vaccination

It is generally well accepted that balanced immune responses between humoral and cell-mediated responses are the desired outcome from vaccination. For this reason, MLVs are often considered the vaccine of choice for veterinary applications where the virus of interest is amenable to robust attenuation [64,65]. An excellent example of this in veterinary medicine is the successful eradication of the rinderpest virus that was largely underpinned by an MLV [66]. The global eradication of variola virus from the human population with Vaccinia (and its derivatives) is arguably the greatest achievement in medicine [67]. While overall a very successful class of vaccines, the disadvantages of MLVs include the potential for reversion to virulence, the interference from pre-existing or maternal immune responses, and the potential lack of suitability for use in animals with compromised immune systems [64]. Furthermore, recombination with the wild-type virus strains can also occur, as has been reported for porcine reproductive and respiratory syndrome MLVs [68]. Despite the success of this approach for many pathogens, the mechanism(s) that underpin the loss of pathogenic potential in the attenuation process remain poorly understood in most cases. This lack of understanding can be highly problematic in instances of vaccine failure, resulting in the loss of efficacy. One potential solution is to move to more defined vaccination formulations, such as mRNA-based formulations, to address these issues.

The ideal mRNA vaccine formulation for veterinary applications would largely mimic the types of innate and adaptive immune responses stimulated by an MLV, while minimizing any potential adverse effects. Of course, this is challenging, as the mRNA formulation must be designed and formulated with these parameters in mind, while the MLV is derived from a pathogen that has evolved largely in response to these complex systems over long periods of time.

### 3.1. Initiation of the Immune Response

Following injection, the mRNA formulation induces localized inflammation, resulting in the recruitment of monocytes, neutrophils, and dendritic cells to the site of injection [69]. After the assimilation of the LNP–mRNA complexes, the monocytes and dendritic cells migrate to the proximal draining lymph node and present the translated antigen to helper CD4+ T-cells via histocompatibility complex class II (MHC-II). The activated helper T-cells subsequently stimulate B-cells, leading to the production of antigen-specific antibodies. The monocytes and dendritic cells can also present the heterologous antigen through the alternate histocompatibility complex class I (MHC-I) pathway to cytotoxic CD8+ T-cells. Once activated, the antigen-specific cytotoxic CD8+ T-cells are able to recognize antigen-expressing cells (e.g., a virus-infected cell presenting viral antigens on its surface) and target them for cytolytic destruction.

As discussed previously, both the mRNA and carrier technologies, such as LNPs, have the capacity to initiate innate immune responses, with many of the key improvements in these technologies aiming to dampen these responses. How the various molecular components of the mRNA can influence the stimulation of innate immune responses has been discussed previously in this review and will not be repeated here.

### 3.2. Humoral Immune Responses

Wu et al. [70] recently compared the immune responses of mice immunized with an LNP–mRNA formulation to mice immunized with a conventional adjuvanted subunit vaccine. Both formulations were based on the SARS-CoV-2 spike protein as the antigen. The mice were immunized twice, 14 days apart, with no differences in antigen-specific IgG detected at day 7 or day 14 after the final immunization. However, at day 21 postimmunization, significantly higher levels of antigen-specific IgG were detected in the mice immunized with the LNP–mRNA formulation. When the day 14 IgG responses were subtyped, the levels of IgG1 were similar between the groups. In contrast, significantly higher levels of IgG2a were detected in the LNP–mRNA-immunized group. The differences in the IgG subclasses were also reflected in the IgG1/IgG2a ratio, with the LNP–mRNA group having a ratio of approximately 1 compared to >10 for the subunit group. The results supported the authors’ conclusion that the LNP–mRNA formulation elicited a balanced Th1/Th2 response, while the subunit formulation elicited a Th2-biased response. The results also suggested the LNP–mRNA formulation would provide higher efficacy over time, as it induced significantly higher virus-neutralizing titres at day 21 compared to the subunit formulation [70]. The study also characterized the cell-mediated immune responses using ELISpot assays, with the results showing significantly more T-cells secreting IFN-γ in the LNP–mRNA-immunized group compared to the subunit-immunized group. These results demonstrated the amount of IFN-γ produced by the T-cells from LNP–mRNA-immunized group was significantly higher [70].

### 3.3. Cell-Mediated Immune Responses

Cell-mediated responses are important for the recognition of cells infected with intracellular pathogens. Lim et al. [71] compared the T-cell responses of three groups of human patients using a whole-blood cytokine assay. The groups were vaccinated with one of the following: the SARS-CoV-2 inactivated vaccine (two doses), a heterologous vaccine (one dose of LNP–mRNA and two doses of a second inactivated vaccine), or an LNP–mRNA. At 21 days postimmunization, no differences were detected in IFN-γ production between any of the groups when overlapping spike protein peptides were used as antagonists. However, the LNP–mRNA and heterologous groups produced significantly more IL-2 than the inactivated-only group at the same timepoint. At 2 to 3 months postimmunization, the LNP–mRNA group produced significantly more IFN-γ and IL-2 compared to the inactivated-only group. The heterologous group was not significantly different to the other groups. The study also evaluated the phenotypes of the T-cells, with the LNP–mRNA stimulating the activation of both CD4+ and CD8+, whilst the vaccination regimes, including the inactivated virus only, activated C4+ T-cells [71]. Overall, the study supported the capacity of the LNP–mRNA formulations to elicit robust cell-mediated immune responses. The results of the study also demonstrated the capacity of an mRNA formulation to elicit superior immune responses to an inactivated vaccine, the type of vaccine that is widely used in veterinary medicine.

### 3.4. Mucosal Immune Responses

Of particular interest to protecting against respiratory pathogens is the induction of robust immune responses at the mucosal surfaces of the upper respiratory tract, as it is typically the site of first contact between the pathogen and the host. Azzi et al. [72] recently reported that the COVID-19 mRNA vaccine BNT162b2 induced strong circulating IgG-neutralizing antibodies in humans (*n* = 60) following intramuscular vaccination. The study compared the levels of antigen-specific IgG and IgA in serum and saliva. While neutralizing antibodies were detected in saliva after the second immunization, the levels were lower than those detected in serum, with the response largely mediated by the secreted IgG rather than the IgA. These results were supported by the results of another study by Nickel et al. [73] that reported no evidence of mucosal immune responses mediated by IgA in human patients vaccinated with BNT162b2 [73].

As it is well recognized that the immune responses at the mucosal surfaces are to some extent linked [74], the results to date suggest that further research is required to improve the mucosal responses from the mRNA-based intramuscular vaccination. Similarly, investigating alternative routes for the delivery of mRNA vaccines may improve the stimulation of immune responses at the desired mucosal surfaces. As an example, Cuburu et al. [75] demonstrated that sublingual immunization was able to elicit systemic and mucosal immune responses in mice. When immunizing sublingually with the model antigen OVA, mice were reported to develop Th1/Th2 immune responses, with T-cell response induced in the lungs [75]. Importantly, sublingual immunization protected the mice from lethal intranasal challenge [75]. Hanson et al. [76] recently reported the use of mucoadhesive polymer-based wafers for the sublingual delivery of a protein antigen. Depending on the relative composition of the carboxymethylcellulose and alginate polymers, the wafers were retained on the mucosa for longer and/or prevented the heat destabilization of the proteinaceous antigen [76]. It would be of interest to evaluate the suitability of this system for the sublingual delivery of mRNA vaccines.

When applying vaccination to many livestock populations, it is rare that its exposure status to the pathogen of interest is known. As such, one important consideration is what impact existing immune responses might have on vaccination. This is a particular concern for MLVs that could be inhibited by pre-existing immune responses. With respect to young animals, it is the potential for maternal antibodies to interfere with the adaptive immune responses to MLV vaccination [77]. As the maternal antibodies decline, a potential window of pathogen susceptibility can occur before the vaccines can stimulate adaptive responses. Using a murine model influenza virus model, Willis et al. [78] demonstrated that an mRNA LNP formulation was able to stimulate stronger protective antibody responses in the presence of maternal antibodies compared to a conventional vaccine. This study suggests that mRNA vaccination may have the capacity to bridge the gap in the protection afforded by maternal antibodies and that elicited by vaccination, thus reducing the period for which young animals are susceptible to disease [78].

## 4. Key Recent Advances in mRNA Vaccine Design and Delivery

### 4.1. Advances in mRNA Design

The impetus provided by the COVID-19 pandemic has resulted in rapid advances in the design, manufacturing, and delivery of mRNA vaccines to address deficiencies. One of the major deficiencies identified during the rollout of mRNA vaccines during the pandemic was the critical requirement to maintain cold transport and storage chains. It is likely that the deployment of mRNA vaccines for veterinary applications will be similarly constrained, if not more so. While mRNAs are generally depicted as linear molecules, this is not the case; the molecules form complex secondary structures. It is the final confirmation of these structures that can determine the half-life of the mRNA molecule and the efficiency of the polypeptide translation. Studies have shown that an optimal secondary structure and codon usage can improve mRNA stability [79,80]. However, it is possible that empirically optimizing these parameters for each candidate mRNA could be required for superior vaccine performance. To address this issue, Zhang et al. [81] developed an artificial intelligence-based algorithm to reconfigure the tertiary structure of mRNA transcripts. The LinearDesign algorithm enables the rapid and simultaneous optimization of the secondary structure and codon usage patterns of the candidate mRNA to improve the mRNA stability and translation. The study reported the successful application of the algorithm to two viral antigens, demonstrating an improved mRNA half-life and enhanced immune responses in mice [81].

### 4.2. Dose-Optimization Strategies for mRNA Vaccines

The number of immunizations required to elicit protective responses, and the need for and the timing of booster doses to maintain protection, are important considerations with respect to the adoption of veterinary vaccines. This can be particularly relevant in extensive production systems, where livestock management practices are grouped together due to the costs associated with mustering highly dispersed livestock populations. In these systems, management interventions, such as vaccination, can only be administered once or twice a year. Similarly, in developing countries and emerging economies where small-holder farming predominates [82] with little or no animal health support, minimizing the number of doses required and reducing the reliance on cold chains is essential to enable adoption. Thus, innovations are required to meet these challenges that conventional approaches to vaccination have not.

Heterologous prime–boost vaccination is recognized as a potentially effect vaccination strategy [83,84,85]. Briefly, this approach is centred on the synergistic use of two or more vaccine platforms. While the exact mechanisms underpinning this phenomenon remain to be fully elucidated, it is believed that the delivery of the same antigenic components via different routes results in the enhanced stimulation of the immune system. Park et al. [86] recently investigated the use of mRNA and subunit vaccines for the influenza virus hemagglutinin glycoprotein in homologous and heterologous prime–boost immunization strategies. With respect to the antibody isotypes, subunit/subunit and subunit/mRNA immunizations produced significantly stronger IgG1 responses compared to the mRNA/subunit- and mRNA/mRNA-immunized groups, whereas the Ig2a responses were significantly higher in the mRNA/subunit- and mRNA/mRNA-immunized groups compared to the subunit/subunit- or subunit/mRNA-immunized groups. Similarly, antibody isotype switching in the mRNA/subunit- and mRNA/mRNA-immunized groups was further reflected by the well-balanced IgG1/Ig2a ratios in these groups compared to the strong IgG1 biases in the subunit/subunit and subunit/mRNA groups. The mRNA/subunit and mRNA/mRNA groups also had higher virus-neutralization titres.

The study went on to directly compare the prime–boost strategies and reported the detection of significantly stronger cell-mediated immune responses in the mRNA/protein- and mRNA/mRNA-immunized groups compared to the protein/protein-dosed group based on enzyme-linked immune-spot assays for the interferon-γ production by splenocytes. However, the number of CD4^+^ T-cells producing interferon-γ were significantly higher in the mRNA/mRNA group compared to the subunit/subunit group, with no detected advantage for the prime/boost strategy. No significant differences were detected between the treatment groups for the C4^+^ T-cells producing either TNF-α or interleukin 2. With respect to the C8^+^ T-cells, significantly more cells with this phenotype expressing interferon-γ were generated by the mRNA/mRNA immunization compared to the subunit/subunit, mRNA/subunit, and subunit/mRNA treatments. While the C8^+^ T-cells expressing TNF-α were detected in all treatments, the percentages of each group were similar, with no significant differences detected. No significant differences were detected for the C8^+^ T-cells expressing interleukin 2. When the mRNA/subunit and subunit/mRNA strategies were compared in a viral-challenge study, a key finding of the study was an improved IgG2a antibody response in the mice primed with the mRNA vaccine and boosted with the subunit vaccine compared to the reverse prime/boost strategy. Similarly, the mice receiving this mRNA/protein vaccination regime had reduced viral titres and inflammation in the challenge experiments. While the prime–boost strategy will need to be further evaluated in other species, the results of this study suggest that it is a promising approach to improve disease control.

The use of prime–boost vaccination strategies in ruminants has received minimal attention. Durel et al. [87] evaluated if the use of two inactivated commercial vaccines targeting neonatal calf diarrhea could be used in a quasiprime–boost strategy through cow immunization. The antigenic components of these vaccines varied in terms of the inactivated viral and bacterial strains used, and the formulations also contained different adjuvants. The study reported the benefits for boosting with a heterologous dose [87]. It remains to be seen if heterologous prime–boost vaccination strategies that include mRNA will be effective in ruminants. However, as mRNA vaccines for livestock species start to emerge, there will be an opportunity to evaluate them in heterologous prime–boost strategies in combination with existing vaccines. There will need to be considerable benefits from this approach for it to gain widespread adoption by livestock industries, as there are likely to be feasibility and practicality issues around the successful administration of two different vaccine technologies.

### 4.3. Optimization of mRNA Delivery

As discussed previously, the use of ionizable LNPs or similar carriers has proven to be a critical component of the success of the current leading mRNA vaccines. The importance of the delivery platform was further illustrated by a recent study by Suzuki et al. [88]. The study designed and compared the capacity of nine novel LNPs to improve the delivery and physical properties of mRNA formulations. The LNPs were based on cyclic-head N-methyl-piperidine with varying lipid tail structures. The lipid tail structures were shown to influence the mRNA encapsulation capacity and immunogenicity of the mRNA-encoded antigen in mouse studies. In vitro reporter gene studies suggested the increased in vivo immunogenicity was correlated with the increased expression in the mRNA-encoded protein due to the enhanced intracellular delivery [88]. Using the lead LNP identified in their study, the authors investigated the suitability of mRNA doses, based on this formulation, to be lyophilized using glucose as a cryoprotectant to improve the dose stability over time at elevated temperatures. After one month, wet and lyophilized doses stored at 5 °C and lyophilized doses stored at 25 °C induced similar antibody responses in the mice compared to the control doses stored at −80 °C. While the storage at 40 °C resulted in reduced immunogenicity, the lyophilized doses elicited higher mean antibody responses compared to the wet formulation, though no significant differences were detected [88]. Interestingly, the characterization of the dose stability under these various conditions suggested the loss of potency was due to the degradation of the mRNA within the nanoparticles, as the LNPs were shown to retain their integrity [88]. The study also demonstrated the LNPs were potent adjuvants, regardless of the immunogen type, by directly comparing the immunogenicity of a protein antigen to an mRNA encoding the same antigen. The study also directly compared the lead LNP–mRNA formulation to the analogous protein antigen adjuvanted by ALUM. The investigation of the specific cell-mediated immune responses used ELISpot assays for the IFN-γ+- and IL4+-expressing cells, with the LNP–mRNA formulation eliciting a significantly higher ratio of IFN-γ+/IL4+ cells compared to the protein base formulation [88]. The mRNA formulation also elicited both IgG2a and IgG1 antibody responses, suggestive of balanced Th1/Th2 immune responses [88]. In comparison, the protein–ALUM formulation elicited a predominantlyIgG2 antibody response, suggestive of a predominately Th2-type response. The study also investigated the biodegradability of their lead formulation with a direct comparison to ionizable lipid MC3-derived LNPs. The LNP–MC3 was readily detectable in the injection-site muscle, lymph nodes, plasma, liver, and spleen for the duration of the experiment (72 h). In comparison, the LNP–L202 formulation was detected in the muscle and lymph nodes for the same period. However, it was only detectable for 24 h in the plasma and spleen, and could not be detected in the liver after 6 h [88]. Based on these results, the authors concluded the LNP–L202 formulation demonstrated excellent biodegradability. The implications of this study in the contact of livestock vaccination are discussed later in this review.

Chen et al. [89] reported a combinatorial approach for ionizable lipids to improve the design of LNPs with novel properties. A key outcome of the study was the identification of an ionizable lipid, iso-A11B5C1, that facilitated high transfection efficacies in muscle cells and reduced the transfection efficiency in the liver and spleen. Interestingly, reduced transfection was also reported with respect to the lymph nodes and immune cells, resulting in reduced humoral responses; however, strong cell-mediated responses were detected [89]. This study highlights that, as the design of LNPs and their components continue to evolve, the application-specific design of mRNAs for optimal efficiency will become more feasible.

### 4.4. Intranasal Vaccination with mRNA

One approach that is used, particularly with MLVs, is intranasal vaccination. Boley et al. [90] compared the capacity of LNP–protein and LNP–mRNA formulations (both the delivery of the spike receptor-binding domain and nucleocapsid proteins) in a SARS-CoV-2 ferret challenge–protection study following intranasal delivery. Each formulation was also evaluated in combination with monosodium urate encapsulated in the LNPs as an adjuvant. The results of the study suggest that the LNP–protein formulation elicited balanced Th1/Th2 immune responses compared to the LNP–mRNA formulation, which elicited a biased antibody-mediated response based on the cytokine expression profiles in various respiratory tissues [90]. Of note in this study was that the mRNA formulation (two mRNAs plus an adjuvant) induced similar serum IgG responses with or without liposome encapsulation. In comparison, the protein formulation (two proteins plus an adjuvant) only induced IgG responses with liposome encapsulation [90]. This study also demonstrated the capacity of multiple mRNAs delivered simultaneously to elicit strong immune responses to both antigens. As will be discussed in the context of the example diseases included in this review, most mRNA vaccines for veterinary applications will need to be multivalent with respect to the antigens included in the final formulations. While not tested by Boley et al. [90], it would be interesting to evaluate the LNP–protein and LNP–mRNA formulations described in the study using a heterologous prime–boost immunization regime via the delivered intranasal route.

### 4.5. Ribosomal Stalling and Ribosomal Frameshifting

Mulroney et al. [91] recently reported that specific sequence motifs within mRNAs containing N1-methylpseudouridine can result in frame +1 ribosomal frameshifting. The consequence of these potential frameshifts is the translation of nontarget polypeptides and the premature termination of translation for the antigen of interest. The frameshifting was attributed to a combination of ribosome stalling and ribosome slippery sequences. Ribosome stalling was linked to reduced translation efficiency due to the use of N1-methylpseudouridine, while ribosome slippery sequences facilitated programmed ribosomal frameshifting, a natural phenomenon frequently used by viruses [92]. Importantly, Mulroney et al. [91] were able to detect the T-cell responses to some of the nontarget polypeptides produced from the frameshifting in mice and humans. The +1 frameshifting effects could be ameliorated by the inclusion of stop codons within the +1 reading frame [91]. The future design of mRNA vaccines will need to consider these elements to minimize or eliminate the translation of nontarget polypeptides to prevent unintended consequences, such as reduced efficacy and/or toxicity. Ideally, this would be a component of emerging mRNA vaccine design algorithms, such as LinearDesign [81].

## 5. Development of mRNA Vaccines for Important Livestock Diseases

The following sections outline the potential application of mRNA vaccination strategies to selected important ruminant diseases. The discussion is framed within the context of existing vaccines (where available) and recent advances in these diseases to highlight the potential use of mRNA-based vaccination to improve their control.

### 5.1. Lumpy Skin Disease

A disease principally of cattle and buffalo, lumpy skin disease (LSD) is caused by the lumpy skin disease virus (LSDV). The LSDV is classified within the genus *Capripoxvirus* of the family *Poxviridae*. The virus has a large double-stranded DNA genome that is approximately 151 kbp in length with a predicted 156 ORFs [93]. Unlike other viruses with large double-stranded DNA genomes, the LSDV and other poxviruses replicate within the cytoplasm of infected cells. Consequently, the viral genome encodes an array of genes involved in transcription to enable the completion of the viral replication cycle [93]. Of interest in the context of mRNA vaccine development is poxviruses, which encode the repertoire of genes required for mRNA synthesis, including CAP enzymes [94,95]. Indeed, the Vaccinia mRNA capping enzymes are widely utilized in the IVT systems used for mRNA production [51].

While the LSDV remains an important limitation for productivity in the countries where it is endemic, it is also of concern across the world, particularly in the Asia–Pacific region, as it has rapidly moved from the countries in northern Africa to Indonesia in recent years. The major mode of LSDV transmission is via arthropods, with mosquitoes and biting midges considered to be the most important vectors [96]. However, direct animal-to-animal transmission has also been reported [97].

Currently, the control of LSDV from a vaccination point of view is dependent on an MLV Neethling strain, where its use is permissible. This vaccine was developed using a South African field strain of the LSDV that was passaged in lamb kidney cells (*n* = 50), followed by the additional passage in embryonated eggs (*n* = 20). While the vaccine is considered safe and effective, localized reactions are reported in many immunized cattle [98]. Morgenstern and Klement [99] reported no adverse effects with the use of the Neethling MLV Neethling LSD vaccine (OBP, South Africa) in dairy cattle (*n* = 21,844) with respect to milk production or mortality across 77 dairy farms in Israel. This vaccine has also been highly effective in controlling the outbreaks of LSDV on the Balkans Peninsula in southeastern Europe [100].

More recently, the development and evaluation of an alternative LSDV MLV strain has been described by Kumar et al. [101]. The prototype LSDV was developed by the serial passage of an Indian isolate of LSDV in Vero cells. The prototype vaccine proved to be safe and efficacious in experimental and field trials. The prototype vaccine was able to confer full protection to immunized cattle from homologous challenge under experimental conditions. There was no evidence of the prototype vaccine eliciting a Neethling response in the trial animals (0%, *n* = 10), and rarely in the field-study animals (0.018%, *n* = 26,940) [101]. The Neethling response is the collective term used to described adverse effects, such as injection-site reaction, very infrequently small-size skin nodules, or transient reduction in milk production yield, associated with vaccination with the Neethling strain. Of interest, this study reported the complete genome sequences for the LSDV strain prior to (defined as passage 0) and after attenuation (passage 50). Mutations were detected in the genes encoding DNA-dependent RNA polymerase, Kelch-like proteins, EEV membrane phosphoglycoprotein, and the CD47-like putative membrane protein. Further research is required to determine if any of these proteins could be LSDV-protective antigens.

The LSDV MLVs have proven to be highly efficient in countries where the LSDV is considered endemic and in controlling outbreaks in other countries where they are permitted. However, current LSDV MLV strains do not enable the differentiation of infected and vaccinated animals (DIVAs). Consequently, if a previously LSDV-free country were to use these vaccines to control an LSDV outbreak, the culling of vaccinated animals is likely to be required to regain a disease-free status. Similarly, the use of MLV vaccines can prove problematic in the final stages of eradication campaigns if DIVA principles cannot be applied to ensure the accurate identification of vaccinated animals.

Inactivated vaccines can enable the application of DIVA principles, as animals will only generate immune responses to the antigens of the structural components of a virus. Briefly, an animal that has been infected by the virus will have immune responses to the antigens of the structural and nonstructural components of the virus. Thus, serological assays detecting antibody responses to nonstructural antigens enable DIVA principles where infected and vaccinated animals test positive and negative, respectively. To enable the potential use of vaccination in regions where the LSDV is not endemic, Matsiela et al. [102] investigated the immunogenicity of an inactivated LSDV formulation that was adjuvanted with Montanide™ Gel 01 PR in rabbits. The formulation was able to elicit strong humoral responses in a dose-dependent manner. The study did not report cell-mediated responses. Thus, how effective this formulation would be in the control of LSDV under field conditions remains to be determined.

The application of mRNA vaccination to the LSDV is likely to require significant research and development. Currently, there is minimal information available on the putative neutralizing or cell-mediated antigens that could be used to design a prototype LSDV-specific mRNA for evaluation as a vaccine [103]. It is plausible that the application of reverse-vaccinology principles using a combination of bioinformatics and immunoinformatics to identify potential antigens could be used for this process. A recent study utilized bioinformatics to predict LSDV epitopes with the aim of designing a theoretical subunit vaccine [104]. The usefulness of these predicted epitopes could be evaluated using samples from naturally infected animals and/or those immunized with the MLV to confirm if these hypothetical antigens could be used to design the ORF component of a prototype mRNA vaccine. Given the complexity of this virus and the potential redundancy of function, it is considered highly likely that multiple antigens would need to be included in any prototype mRNA vaccine.

Moreover, for an mRNA vaccine for LSDV to enable eradication programs in endemic countries, or effective containment/control/elimination in nonendemic countries, the formulation would need to facilitate the application of DIVA principles. Without DIVA capabilities, it may be necessary to eliminate vaccinated animals to move to or regain an LSDV-free status. While the economic impacts of this approach could be tolerable in countries with developed economies, it could be subject to scrutiny with respect to ethical grounds. The approach would also be undesirable given the potential ongoing economic impacts due to the time required to replace euthanized animals. In developing countries, such an approach would be catastrophic, as bovines can represent a considerable proportion of the wealth within the affected communities. The loss of a single animal can have a dramatic effect on the prospects of the impacted family. The availability of an LSDV DIVA vaccine supported by appropriate diagnostics would enable the effective control of the disease without the need for slaughter. For countries with major cattle industries that have a strong export focus, an LSDV DIVA vaccine could enable the control of outbreaks and accelerate the move back to a disease-free status. Similarly, in developing countries, an LSDV DIVA vaccine would minimize the local impacts of the disease and aid in the design and successful implementation of eradication programs.

Current LSDV MLV strains do not support the application of DIVA principles. Chervyakova et al. [105] developed a prototype MLV LSDV through the disruption of four genes within the genome of the strain Dermatitis nodulares/2016/Atyrau/KZ. The immunization and subsequent challenge of cattle (*n* = 8) suggested the prototype vaccine protected the animals from the clinical signs of LSD. While not examined in the study, the use of an MLV vaccine with gene disruption may also have the capacity for the vaccine to facilitate the application of DIVA principles. The disrupted genes were selected based on their roles as virulence factors in the parental LSDV strain [105]. The study did not report any antigen-specific responses to the deleted gene products. It is likely that, as part of the normal LSDV infection cycle, affected animals would develop antibody responses to at least one, if not all four, of the deleted LSDV proteins. As such, a serological assay based on these polypeptides would enable the identification and differentiation of vaccinated animals from those infected with field strains of the virus.

This theoretical approach highlights a potential limitation of many current DIVA strategies, in that the result of interest, i.e., vaccinated only, is a negative result in the discriminating assay. Similarly, given the paucity of information available on LSDV, the possibility of circulating field strains also lacking potential DIVA antigens cannot be excluded. For example, the eradication of bovine herpesvirus 1 in some jurisdictions has used DIVA principles based on low-virulence glycoprotein E negative-marker vaccine strains. Interstrain recombination between these vaccine strains and virulent wild-type viruses in both experimental and field studies has been reported [106,107]. Clearly, the transfer of the DIVA phenotype to a virulent strain would negate the use of any DIVA diagnostics.

Thus, a DIVA system whereby the key outcome is defined by a positive result would be highly desirable. This could be achieved in several ways. A heterologous antigen could be used, such as green fluorescent protein, where the host of interest is extremely unlikely to have been exposed to the antigen of choice, and therefore would not have pre-existing immune responses to it. Of course, depending on the selected antigen and its source, there may be ethical or moral objections to its use in food-producing animals. Alternatively, it may be feasible to design a synthetic antigen that is designed and constructed specifically for the DIVA application of interest. Such an approach could enable the simultaneous application of DIVA principles against multiple pathogens, if required. Importantly, such an antigen would make the key phenotype of interest in any supporting diagnostics a positive result.

There have been no reports published to date describing the application of mRNA vaccine technologies to any of the animal poxviruses or enabling DIVA-based vaccination principles. What is clear, however, is that any LSDV mRNA vaccine will need to be multivalent in nature. One way to achieve this from a single mRNA molecule is to construct the ORF by fusing multiple LSDV antigen genes. If it is desirable to have each of the LSDV antigens present as individual polypeptides, the respective genes could be separated by segments encoding for 2A peptide motifs from the picornavirus family [108]. The 2A peptides motifs are an intriguing strategy used by members of the picornavirus family to enable the production of multiple polypeptides from an ORF via a mechanism that is believed to involve ribosomal pausing/skipping. It is possible that the order in which the LSDV genes are placed in the synthetic ORF will need to be empirically determined for optimal antigen production. Importantly, the flexible nature of producing candidate mRNAs suggests the efficient evaluation of this multifactorial design would be highly feasible.

The critical impediment to the development of an effective LSDV mRNA vaccine is the lack of information available on protective antigens and/or epitopes that could be used to design the ORF component of any prototype mRNA vaccine. Kar et al. [104] reported a computation-based approach to identify putative LSDV antigens. Analysis of the LSDV proteome identified 32 putative structural/surface proteins. A subset of these antigenic polypeptides (*n* = 4) was predicted to be antigenic and highly conserved across the 26 LSDV isolates examined. The study went on to design a theoretical fusion polypeptide based on the four antigen coding sequences. Further bioinformatic analyses suggested the theoretical fusion polypeptide contained cytotoxic lymphocyte and B-cell epitopes, suggesting that it has the potential to elicit cell-mediated and antibody responses in vivo [104]. It remains to be determined if an antigen- and/or epitope-minimization approach will be successful for complex viruses like LSDV. Rather, it may be more effective to include a wide repertoire of potential immunogens to underpin vaccine efficacy.

Within the poxvirus family, there is evidence of immunological cross-protection between viruses within the same genus. As an example, the members of the *Capripoxvirus* genus LSDV, goat poxvirus and sheep poxvirus, are known to elicit cross-protective immune responses [109]. Similarly, in the genus *Orthopoxvirus*, Vaccinia, cowpox virus, monkeypox virus, and variola (smallpox) virus generate cross-protective immune responses [110]. As a consequence of the importance of the orthopoxviruses to human medicine, there is a larger body of scientific literature underpinning vaccine development. However, even for the orthopoxviruses where the variola virus has been eradicated, the antigenic components underpinning immunological protection are poorly understood. Shchelkunov and Shchelkunova [111] summarized the immunological responses of vaccinated humans to vaccina polypeptides, with no antigens identified that 100% of vaccinees responded to immunologically.

As suggested previously, the key challenge that needs to be met for the development of an LSDV mRNA vaccine is the identification of the protective antigenic components of the virus. There are several strategies that could be used to achieve this. Firstly, the prototype LSDV MLV described by Chervyakova et al. [105] could be used as the basis of an antigen discovery tool. The total RNA from bovine cells infected with LSDV could be utilized to generate a library of cDNA transcripts cloned under the control of the T7 promoter. The cDNA library could then be used to generate transcribed copies of the encoded RNAs to enable the evaluation of transcribed polypeptides as potential antigens in an approach that would be similar to the early mRNA cancer vaccine studies for either in vitro or in vivo antigen studies [20]. The approach could be refined to include the evaluation of select sets of putative antigens to define the protective repertoire required for LSDV. Importantly, the inclusion of whole antigens would reduce the risk of vaccine failure from MHC variations in the population of interest, not recognizing the peptide antigens identified through BoLA bioinformatics analyses. Complete antigens are also more likely to elicit immunological responses to spatial epitopes, therefore potentially increasing vaccine efficacy.

A further refinement to enable antigen discovery would be to develop an infectious clone system for LSDV. Infectious clones have been described for other members of the poxvirus family, including Vaccinia, cowpox virus, and horsepox virus [112,113,114]. Infectious clone systems are powerful tools that can enable an in-depth understanding of viral gene function and host–virus gene interactions. Central to this is identifying the viral genes that are essential and nonessential for the in vitro replication of the viruses of interest. Gene-editing technologies could be used to specifically target candidate genes [115]. Alternatively, random insertions throughout the cloned genome with transposons can enable the assessment of coding and noncoding regions of the viral genome in replication [116]. This knowledge can then be used to identify potential virulence factors and those genes encoding antigens that have the capacity to elicit protective immune responses, with the latter group of genes and their respective polypeptides then used to underpin the design of mRNA-based vaccination strategies.

Recent advances in the development of vaccines for the zoonotic orthopoxvirus, monkeypox virus, also suggest that the development of an LSDV mRNA vaccine is highly feasible. Sang et al. [117] reported the evaluation of two quadrivalent mRNA formulations for monkeypox virus. The formulations stimulated strong antibody and cell-mediated immune responses in mice following two intramuscular injections. The antibody responses were specific to the mRNA-encoded antigens and potent neutralizing antibodies to Vaccinia virus [117]. Importantly, the quadrivalent formulations were protective in a mouse–Vaccinia challenge model [117].

Further support for a multivalent poxvirus mRNA vaccination strategy was reported in another monkeypox virus study. Zeng et al. [118] reported the development and evaluation of two multivalent monkeypox virus mRNA vaccine formulations that contained either four or six mRNAs encoding viral antigens. Of particular interest in this study were the two approaches used to produce the candidate mRNA formulations. In the first approach, the mRNAs were produced by IVT, followed by LNP encapsulation, then combining the individual LNP–mRNAs into the multivalent doses. The second approach was combining the IVT templates prior to the mRNA production via IVT, followed by LNP encapsulation. Interestingly, when the mRNAs were produced in a single IVT reaction, differences in the proportion of the mRNAs produced in the quadrivalent and hexavalent formulations were detected, suggesting different transcription rates between the mRNAs [118]. While these differences were not statistically significant in the study, the monitoring of this phenomenon for other vaccine targets may be required to ensure the adequate representation of all mRNAs in the final formulation. In mice, both formulations induced similar Vaccinia-neutralization antibody titres and protected mice from lethal virus challenge [118], while the in vitro cell-mediated responses were significantly higher in hexavalent mRNA formulations compared to the quadrivalent mRNA formulation [118]. Apart from the protective capacities of these formulations, the demonstration that multivalent mRNA vaccines can be produced by the mixing of the IVT templates is an important advancement in this field, as it streamlines the production pipeline for these types of formulations. Such production refinements are likely to be essential to minimize the cost per dose to underpin the adoption of mRNA vaccines for veterinary applications.

### 5.2. Rift Valley Fever

The arbovirus, rift valley fever virus (RVFV), is a member of the genus *Phlebovirus* of the family *Bunyaviridea*. The RVFV mainly affects domestic ruminants in the countries of sub-Saharan Africa and those in the eastern and southern regions of the African continent, including Madagascar [119]. The RVFV is a potential zoonosis, transmitted to humans via contact with fluids and/or tissues from infected animals; vectorborne transmission is also possible. The RVFV is potentially a virus of global concern. Whilst the current distribution of the RVFV is limited, competent vectors are widely distributed, and as changes in the global climate continue to destabilize current biological systems, there is an increasing risk of this pathogen expanding its geographic distribution [120].

Members of the genus *Phlebovirus* are characterized by segmented single-stranded RNA antisense genomes. The tripartite genomic segments are named according to their relative sizes, large, medium, and small. The large genome segment encodes the viral RNA-dependent RNA polymerase, required for the production of viral mRNA and nascent viral genomic RNA [121]. The small segment encodes the nucleoprotein and nonstructural protein. The nonstructural protein is considered a virulence factor that inters with multiple antiviral functions in infected cells [122]. The medium genomic segment is the most important from a vaccine development point of view, as it encodes the key protective antigens, the surface proteins glycoprotein n and glycoprotein c. The translation of the medium segment transcript involves ribosomal scanning that results in the production of multiple proteins, depending on which initiation codon is utilized. This process results in the production of two nonstructural proteins, 98 kDa and 14 kDa, from the first and second start codons, respectively [123]. The two glycoproteins, glycoprotein n and glycoprotein c, are produced from the fourth start codon [123]. Whether the remaining start codons result in the translation of additional functional polypeptides remains to be determined.

Given the importance of the RVFV to both veterinary and human medicine, there is a considerable body of literature describing the application of a wide variety of vaccine technologies to solve this issue. It is beyond the scope of this review to evaluate these approaches; as a result, only those with the potential to directly impact the development of an RVFV mRNA vaccine will be assessed here. For a more comprehensive assessment of RVF vaccination, the reader is directed to a recent review on the subject [122]. The aspects of prior vaccine development studies of relevance to the development of an mRNA vaccine for veterinary applications are briefly summarized below.

As with many viral diseases affecting veterinary species, MLVs were the first vaccines to be developed for the RVFV. The MLV strain Smithburn was attenuated by serial passage in the brains of mice. In addition, the RVFV ZH548 strain was passaged in the human fibroblast cell line MRC-5 in the presence of the antimetabolite drug 5-fluorouracil. Following plaque purification and amplification, the progeny strain MP-12 was selected for further evaluation based on its attenuation profile in mice [124]. Since its development and subsequent production under Good Manufacturing Practice conditions, the MP-12 has been tested in various model species, livestock species, and safety/immunogenetic clinical trials in humans, as reviewed by Ikegami [125]. The RVF Clone 13 isolate has also been utilized as an MLV. Clone 13 was isolated from a human RVF case; it is a naturally occurring mutant that has a partial deletion in the nonstructural protein ORF within the S genomic segment [126]. RVF Clone 13 has been reported to provide protection against the virulence challenge of sheep and cattle [127,128]. While this has an improved safety profile compared to other RVFV MLVs, some concerns remain around its use in pregnant ewes [129].

Using an MP-12 reverse genetics system, Ly et al. [130] constructed an MP-12 variant with silent mutations distributed every 50 nucleotides within the coding regions of the three genomic segments to investigate the potential for the RVFV to undergo interspecies genomic reassortment with other phleboviruses that might be simultaneously present in insect vectors. While interspecies reassortment was not detected, control infections did identify random genomic segment exchanges between the parent MP12 and the variant virus (MP12-GM50). When the protective efficacy of the MP12-GM50 was evaluated in mice, the vaccinated mice were 100% protected from a lethal RVFV challenge from a single intramuscular dose [131]. The study also reported that the reassortment of the genomic segments of the MP12-GM50 with a pathogenic strain of the RVFV resulted in the reduced virulence in mice, suggesting a multigenomic segment basis of attenuation, and that, if this recombination were to occur in the field, there was a low risk of increasing virulence in the resulting progeny [131]. The results also suggested that the attenuation of the MP12-GM50 was likely to be stable, again minimizing the risk of reversion to virulence in the field. As the nucleotide substitutions with the MP12-GM50 are not present in the field strains of the RVFV, the introduced mutations would also enable the molecular-based monitoring of the vaccine in the field.

Multiple viral-vectored prototype vaccines, including LSDV, adenovirus, Newcastle disease virus, Vaccinia virus, equine herpesvirus 1, capripox, and rabies virus (inactivated), have been developed to target the RVFV [122]. Most of these viral-vectored approaches were underpinned by the delivery of one or both glycoprotein n and glycoprotein c ORFs as heterologous antigens. Most recently, a nonreplicating simian adenovirus-vector (ChAdOx1) encoding codon-optimized transgene for glycoprotein n and glycoprotein c was evaluated in human phase I clinical trials [132]. The prototype vaccine was well tolerated and induced high-titre RVFV-neutralizing antibodies.

DNA-based subunit vaccines have also focused on the RVFV glycoproteins in various forms, while some studies report the inclusion of other RVFV ORFs to improve the efficacy [122]. Of note within the DNA vaccine studies is Bhardwaj et al. [133], who reported the evaluation of DNA vaccine constructs that encoded truncated and soluble forms of the glycoprotein n which fused the three copies of the complement protein C3d as a molecular adjuvant to improve the specific immunological responses to the RVFV antigen. The molecular adjuvanted glycoprotein n construct was able to elicit similar antibody responses compared to an alphavirus replicon encoding the same antigen and MLV MP12. Interestingly, only the alphavirus replicon and MLV MP12 induced detectable cell-mediated responses. However, the DNA vaccine encoding the C3d adjuvanted glycoprotein n protected 100% of mice from the RVFV lethal challenge compared to 80% protection in those immunized with the glycoprotein n DNA vaccine [133].

Prototype RVFV subunit vaccines have also focused on the delivery of the viral glycoproteins. Multiple studies have utilized insect-derived expression systems to express various truncated forms of the RVFV glycoproteins combined with different adjuvants. The ectodomain of glycoprotein n has been evaluated alone or in combination with complete glycoprotein c emulsified with various water-in-oil adjuvants. Sheep immunized subcutaneously with a single dose of glycoprotein n ectodomain and challenged with virulent RVFV at day 19 were afforded limited protection from virulent RVFV challenge. However, in the same study, sheep immunized with the Newcastle disease virus-vectored glycoprotein n ectodomain and glycoprotein c were fully protected from RVFV challenge [134].

The immunization of sheep with the ectodomain of glycoprotein n- and glycoprotein c-adjuvanted Montanide™ ISA25 elicited low-titre RVFV-neutralizing antibodies after one dose [135]. Following the administration of a booster dose with the same formulation, the neutralization titres increased up to 32-fold, with the neutralizing antibodies persisting for at least 328 days postvaccination. This study also reported the successful application of the RVFV nucleocapsid protein for differentiating infected from vaccinated animals [135]. A later study confirmed that this formulation conferred completed protection to sheep from heterologous challenge with the virulent RVFV 28 days after the administration of the booster vaccination [136].

Recently, Wilson et al. [137] reported the evaluation of an RVFV subunit vaccine formulated with the ectodomain of the glycoprotein n and/or complete glycoprotein c emulsified with the oil-in-water adjuvant Montanide™ ISA-25 in cattle immunization studies. The study demonstrated that two doses of glycoprotein n with glycoprotein and two doses of glycoprotein n given three weeks apart and fourteen days prior to the challenge both provided complete protection from the challenge with the virulent RVFV. Similarly, a single dose of the glycoprotein n/glycoprotein c formulation administered 35 days prior to the challenge also provided complete protection from the challenge. However, a single dose of the glycoprotein n formulation administered 14 days prior to the challenge was not protective.

Collectively, these prior studies of DNA vaccines and subunit vaccines report excellent levels of safety and efficacy from the virulent RVFV challenge using the various forms of the glycoprotein n and the complete glycoprotein c as antigens. Clearly, these antigens would be the logical components upon which the development of an RVFV mRNA vaccine could be based. Depending on the degree of host optimization required, it may be feasible to utilize the same viral components in the mRNA vaccine in both livestock and human immunization programs. However, for the livestock vaccine, the implementation of DIVA principles will be required to underpin successful eradication and control programs in endemic and exotic outbreak scenarios, respectively.

An mRNA vaccine would also enable the rapid redesign of the encoded antigens to match the circulating strains of the RVFV if or when required. While protection from heterologous RVFV challenge has been reported for some subunit vaccines [136], it is likely that the best protection from disease will be afforded by matching vaccine components with circulating field strains. Whether there is sufficient variation in field strains to warrant this approach is yet to be determined.

As suggested previously, the logical starting point for an RVFV mRNA vaccine would be the glycoprotein n and glycoprotein c ORFs that have been shown to stimulate protective immune responses when administered via various delivery systems in multiple species. The strategies suggested previously for the development of an LSDV mRNA vaccine with multivalency and DIVA capabilities could also be used to improve the control of the RVFV.

### 5.3. Peste des Petits Ruminants

Peste des petits ruminants virus (PPRV), species name *Small ruminant morbillivirus*, is a member of the genus *Morbillivirus*, of the subfamily *Orthoparamyxovirinae*, and within the family *Paramyxoviridae*. The disease, peste des petits ruminants, associated with this virus, PRR, is typically clinically overt in small ruminants, sheep, and goats, and subclinical in larger ruminants, such as cattle and buffalo [138]. Of the susceptible animals, goats are typically recognized as being the most severely affected species by PPRV infection [139]. Interestingly, the results of a recent serological survey suggest that buffalo are highly susceptible to the PPRV, with sheep (*n* = 100), cattle (*n* = 190), and buffalo (*n* = 144) reported to have been seropositive at 16%, 4%, and 42%, respectively [140]. The global importance of the PPRV is demonstrated by the PPR Global Eradication Strategy that aims to eliminate the virus by 2030 [141].

While there are four recognized genetic lineages of the PPRV, importantly, from a vaccine perspective, they are considered as the same serogroup. While reviewing the molecular epidemiology of the PPRV, Dundon et al. [142] reported several interesting observations with respect to the four lineages. The PRRV lineages I to III were first identified across the African continent, while lineage IV was predominant in the Middle East and Asia. Lineage I strains have not been detected in Africa for over 20 years. In contrast, the evidence reported suggests the lineage IV strains are circulating across Africa [142]. More recently, the detection of lineage IV strains in several countries in West Africa confirm the continuation of this phenomenon [143]. The driver(s) of these observations are unknown, with variations in the virulence, vaccine susceptibility, transmissibility, and/or environmental stability of lineage IV viruses hypothesized as possibly contributing to this phenomenon [142].

The cross-lineage efficacy of the two commonly utilized vaccine strains, Nigeria/75/1 and Sungri/96, are from lineage II and lineage IV, respectively. Hodgson et al. [144] confirmed the cross-lineage capacity of the PRRV vaccine strains. Quantitative differences were detected in the immunological responses elicited by the vaccines. As an example, the vaccination of goats with Nigeria/75/1 resulted in higher serological responses compared to Sungri/96. Sungri/96 vaccination elicited stronger cell-mediated responses (proliferation and interferon production) following the in vitro stimulation of peripheral blood mononuclear cells with a heat-inactivated virus. Both vaccine strains elicited similar numbers of CD8+ T-cells, while Sungri/96 elicited higher numbers of CD4+ T-cells. The separate groups of vaccinated goats in the study were challenged with either an isolate from lineages I, III, and IV, or one of two lineage II isolates. The study reported complete protection from clinical signs for both vaccines, regardless of the challenge strain, confirming the cross-lineage protective capacity of the Nigeria/75/1 and Sungri/96 vaccine strains [144]. The results of this study suggest that the protective capacities of the MLV vaccines are unlikely to have played a role in the emergence of lineage IV viruses globally. The study reported an absence of clinical signs in the challenged animals, with no vaccinated animals testing positive for PPRV challenge viruses by quantitative reverse transcriptase PCR analyses of blood [144]. It should be noted that the virus detection results for the vaccinated animals challenged with the lineage IV strain could not be reported due to the lack of appropriate samples. However, there is the potential for the vaccine efficacy to vary under field conditions.

More recently, potential immunological mismatches have been reported between vaccine and field strains. As an example, Pakistan has utilized the Nigeria 75/1 lineage II vaccine strain for the control of the PRRV; however, the dominant field strains in the country are from lineage IV. Aziz et al. [145] partially characterized the circulating field strains of the PRRV in Pakistan by the sequencing of the coding regions for the nucleocapsid, fusion, and hemagglutinin proteins. In a subsequent evaluation using immunoinformatics, amino acid substitutions were identified in some of the predicted epitopes of the three proteins in the lineage IV field strains. While the importance of these variations are yet to be experimentally validated, the authors suggest that potential epitope mismatch between the lineage II vaccine strain and the lineage IV field strains could be partially to blame for the difficulties in controlling the PRRV in Pakistan [145].

As with other eradication campaigns that are largely dependent on the widespread use of MLVs, there is an unmet need for a DIVA-enabling vaccine for the PPRV. To address this issue, Selvaraj et al. [146] developed a reverse genetics system for the PRRV MLV Sungri/96 strain. Using reverse genetics systems for the PRRV MLV strains (Sungri/96 and Nigeria 75/1), the study replaced the highly variable region in the nucleoprotein (region-IV, aa 406–525) with the corresponding motif from dolphin morbillivirus. Goat immunization and challenge studies demonstrated that the modifications made to the nucleoprotein did not affect the capacity of the MLV strains to protect goats from the PRRV challenge [146]. Importantly, the heterologous nucleoprotein motif enabled the differentiation of vaccinated animals from infected animals using specific ELISA kits. This study is interesting from several points of view. Firstly, it potentially enables the application of DIVA principles without compromising the capacity of the MLV to elicit protective immune responses by using the homologous component of another virus. It also switches a common DIVA paradigm discussed previously, where the identification of a vaccinated animal yields a negative result. While, conceptually, a negative result is as equally valid as a positive result, pragmatically, a positive result for the outcome of interest, vaccinated, is likely to be more easily interpretable, particularly in challenging field conditions or low-technology settings. The study also demonstrates the concept of using heterologous antigens for DIVA applications. In this case, the homologous domain from dolphin morbillivirus was selected, as it preserves the function of the PRRV nucleoprotein and is underpinned by the assumption that there are currently no morbilliviruses known with such a high identity in this domain that infect goats and sheep. Clearly, if these vaccines were to be widely adopted, ongoing surveillance would be required to ensure the early detection of the emergence of either new variants of PRRV strains or morbilliviruses that are more closely related to the dolphin morbillivirus virus, which has the capacity to infect goats, sheep, or other ruminants.

The current PPRV MLVs induce cross-lineage protection, and this is reported to last for at least three years postvaccination [147]. As with other members of the *Paramyxoviridae* family, the fusion and hemagglutinin proteins are the key protective antigens from a vaccine development perspective. Several alternative vaccine platform technologies using the PRRV fusion and hemagglutinin proteins have been reported. For example, PPRV replicon vector DNA vaccines encoding either the PPRV fusion or hemagglutinin proteins are reported to elicit humoral and cellular responses in a mouse model [148,149]. Similarly, plasmid-based DNA vaccination encoding an anti-idiotype antibody for the hemagglutinin protein was able to induce neutralizing antibodies and in vitro T-cell responses in sheep. However, these studies did not test the capacity of these formulations to protect animals from PPRV challenge.

There are reports that have evaluated the use of the PRRV fusion and hemagglutinin ORFs across different vaccination platforms. As an example, Li et al. [60] reported on the capacity of a PPRV subunit vaccine that utilized the hemagglutinin fused to the bacterial protein ferritin to induce immune responses in mice. When expressed in bacterial (*Escherichia coli*) or baculovirus expression systems, the hemagglutinin–ferritin polypeptide self-assembled into nanoparticles that improved the efficacy of dendritic cell uptake. The sera harvested from mice immunized with either polypeptide were able to neutralize PPRV under the appropriate conditions.

There are also numerous reports describing the development of a viral-vectored vaccine for the PPRV. While proposing the use of an adenovirus vector for the PPRV, Rojas et al. [147] recently summarized the virus vectors tested for the delivery of the fusion and hemagglutinin proteins; as such, this will not be repeated here, other than to state that PPRV-vectored vaccines using vaccinia virus, fowlpox virus, capripox virus, bovine herpesvirus 4, Newcastle disease virus, human adenovirus, and canine adenovirus have all been investigated [147].

A clear starting point for the development of a PPRV mRNA vaccine would be development of optimal plasmid constructs to produce mRNAs encoding the fusion and hemagglutinin proteins. As the most effective PPRV subunit and vectored vaccines have utilized both of these proteins, it is likely that the development of an mRNA that produces both of these proteins would be the most effective strategy. Given the flexibility of the mRNA postproduction formulation, it may also be feasible to efficiently alter the composition of these formulations to match the circulating strains within a particular country or region. A PPRV mRNA vaccine formulation will also need to enable the application of DIVA principles, ideally through the positive detection of vaccinated animals, as discussed previously. While the use of a homologous antigen from a related virus in a phylogenetically distinct host has been shown to be feasible, it does require caution with respect to the potential emergence of uncharacterized viruses that could interfere with the process. Alternate strategies may be required to prevent this, such as using an antigen from a heterologous system, like green fluorescent protein. Alternatively, as immunological prediction algorithms improve and are combined with artificial intelligence, the design of unique antigens for DIVA applications should be plausible. The use of an mRNA-based delivery system would enable the testing of the compatibility of these DIVA antigens in combination with the protective antigens of the pathogen of interest, such as those for PRRV, to ensure that they do not compromise protective immune responses.

### 5.4. East Coast Fever

East coast fever (ECF) is a tickborne disease that is caused by the apicomplexan organism, *Theileria parva* [150]. The disease has a major economic impact on cattle productivity in sub-Saharan Africa, where it is associated with the death of over one million cattle every year, with the total economic impact estimated at USD 596 M [151]. Currently, the control of this disease can be implemented using one of two strategies. The first is to minimize the risk of transmission by reducing the number of tick vectors by applying chemical treatments known as acaracides. An antiprotozoal treatment, buparvaquone, can also be used to treat infected cattle. The effectiveness and long-term efficacy of these approaches can be reduced by the development of resistance to acaracides and buparvaquone by the tick vector and the parasite, respectively. The prevention of ECF relies on a strategy referred to as “infection and treatment” (ITM). ITM is the simultaneous infection with live *T. parva* sporozoites and treatment with the long-lasting antimicrobial oxytetracycline. While ITM can be highly effective, it has several limitations, including the consistency and scalability of manufacturing, the requirement for cold-storage chains, and geographic-based antigenic variations in *T. parva* strains. As ITM elicits immunological responses that provide long-term protection from ECF, it supports the hypothesis that a safe and effective vaccine would improve the control of this disease.

One antigen, p67, the major surface antigen of *T. parva*, has been identified with the capacity to underpin the development of an effective ECF vaccine [152]. Studies have suggested that two discontinuous epitopes within an 80 amino acid domain from the C-terminus of p67, referred to as p67C, are crucial for protective immune responses. Musoke et al. [153] reported that three doses of p67C afforded similar levels of protection, 50% efficacy at LD70, to cattle from ECF challenge, as with the whole p67 antigen. However, reducing the number of immunizations to two resulted in a dramatic loss of protective efficacy to 25% at LD70 [154]. Lacasta et al. [155] reported strong antibody and CD4 T-cell responses to p67C in cattle following coimmunization with 450 µg p67C per dose delivered via two nanoparticle technologies. The level of protection afforded was the highest reported for the p67C antigen to date, with 53% efficacy at LD93 [155]. More recently, efficacy approaching 50% against sporozoite challenge was reported with 140 µg p67C per dose delivered using a computationally designed nanoparticle [156]. Further supporting the potential of the p67C is the high degree of conservation of this antigen. Musoke et al. [153] reported that, for otherwise genetically diverse field strains of *T. parva*, p67 was 100% identical. Collectively, these studies suggest that the p67C or the complete p67 coding sequences would be an excellent starting point for the development of the design of an mRNA vaccine for ECF.

Kolakowski et al. [157] recently reviewed ECF vaccine development, highlighting three aspects of *T. parva* biology that have been largely neglected. The first of these is the poor understanding of host cell invasion and the potential importance that post-translation modifications could play in this process. The recent identification of glycotransferases in *T. parva* RNAseq datasets suggest that the existing paradigms suggesting that this parasite lacks the capacity for glycosylation may be incorrect. As the glycosylation of proteins and lipids are integral for the attachment and invasion process of other Apicomplexan parasites, it might be expected that *T. parva* would also have this capacity [158].

An mRNA vaccine has the potential to address this deficiency for proteinaceous antigens that may undergo post-translational modifications, such as glycosylation. With an appropriate design, the expressed antigen can be directed to the endoplasmic reticulum to undergo the addition of glycan moieties. Clearly, the capacity of *T. parva* to complete the post-translational modification of potential antigens will need to be confirmed before prospective antigens are directed into these pathways. Similarly, the potential for unintended post-translational modification, such as the glycosylation of cryptic motifs in the pathogen polypeptide that might mask or sterically hinder the recognition of protective epitopes, will require investigation.

The second knowledge gap identified was the lack of understanding of the *T. parva* proteomes of each live stage, and therefore the potential repertoire of candidate antigens [157]. Using comparative transcriptomes and bioinformatic analyses, Atchou et al. [159] identified several novel candidate antigens. To validate the pipeline, two candidates encoded by genes TP04_0076 and TP04_0640 were selected for further experimentation. These genes selected as the encoded polypeptides were likely to encode membrane proteins and were predicted to have high immunogenic potential. The antigenicity of the encoded polypeptides was confirmed using a bacterial-expressed polypeptide (TP04_0076) and synthesized peptides (one for TP04_0076 and two TP04_0640) with sera samples from cattle known to be serologically positive to sera *T. parva* [159]. No published studies were identified that evaluated any of these putative antigens in cattle protection studies.

The flexibility of an mRNA vaccine platform would be ideal to address this knowledge gap. The capacity to efficiently exchange ORFs encoding candidate antigens would enable the construction of an mRNA antigen library for *T. parva*. Furthermore, the availability of such a library would enable the efficient multiplexed evaluation of candidate antigens. This could also include p67 or p67C, with new antigens potentially improving the protection afforded by this well-characterized antigen. Similarly, the flexibility afforded in mRNA vaccine formulation would accelerate the evaluation of a p67 mRNA in coimmunization studies with other mRNAs encoding candidate antigens, such as TP04_0076 and TP04_0640.

The third knowledge gap identified was the poor understanding of what constitutes a protective immune response [157]. It is generally accepted that strong cell-mediated responses play crucial roles in providing protection from and reducing the severity of *T. parva*-associated disease. The obvious consequence of this knowledge gap is that optimal immune response from vaccination is also unknown. As a result, the only way to evaluate the protective efficacy, particularly for a complex pathogen like *T. parva*, is to undertake challenge studies in the host of interest. While a protection study is a desirable final step of most vaccine development programs, it is not an effective candidate screening strategy. Unavoidably, including a challenge component will increase the time, complexity and risk profile, and resources required to complete such studies. In particular, for *T. parva*, complexity and risk profiles are important, as variations in the challenge sporozoite dose and/or natural variation in host susceptibility can be important. These variables may impact the efficacy estimates, making it problematic to compare between trials over time. In contrast, improved knowledge of what constitutes a protective response can be used to replace in vivo challenges with in vitro assays. In most instances, this will accelerate research programs and utilize fewer resources. This approach also has the capacity to improve animal welfare outcomes through improved experimental design, where only animals with optimal immune responses are challenged, and are therefore likely to be protected. Moreover, mRNA vaccines are known to elicit strong and balanced immune responses [70]. This suggests that the development of a p67- or p67C-based mRNA vaccine would be a worthwhile undertaking. As reported by Lacasta et al. [155], and described above, the p67C antigen has the capacity to elicit protective humoral and cell-mediated immune responses, depending on how it is presented to the immune system, identifying it as a prime candidate antigen for mRNA vaccine development.

### 5.5. Bovine Respiratory Disease

Globally, bovine respiratory disease (BRD) is the most important cause of morbidity and mortality of intensively finished cattle. It has been estimated that BRD results in annual losses of USD 4.97 billion in the US alone [160]. The development of BRD is complex, and there are a multitude of factors that can influence the risk of an animal being diagnosed with BRD. A well-excepted model for BRD development is cattle arriving at the feedlot having suboptimal immune functions because of multiple stressors. At the same time, cattle may be exposed to or mixed with cattle from different sources, thus increasing the risk of viral transmission; in combination with immune suppression, the viral infection(s) may be more severe, and in some instances, further decrease immune function. These factors render the animals highly susceptible to secondary bacterial infections that can progress to BRD [65], while severe cases of BRD can lead to the death of the affected animal. Other cattle will be diagnosed with BRD and undergo treatment with antimicrobials. Typically, cattle are most at risk of BRD in the first 14 to 28 days after feedlot induction, with the majority of BRD cases in the first 50 days on feed [161].

Epidemiological studies have demonstrated that prior exposure to pathogens associated with BRD can reduce the risk of feedlot cattle being treated for BRD, suggesting that vaccination has an important role to play in reducing the negative impacts of this disease [162,163]. Consequently, significant investments have been made to develop and register vaccines for the major viral and bacterial pathogens associated with BRD, with hundreds of vaccines developed for the feedlot industries in North America [164,165]. Despite the availability of a wide range of vaccines of varying types, the prevention of BRD has remained a continuing challenge for affected industries.

Four factors make BRD a particularly difficult target from a vaccine development perspective. The first is the complex aetiology of BRD, where complex interactions, including animal management, environmental conditions, animal genetics, and pathogen exposure, increase the risk of disease. Secondly, the wide repertoire of viral and bacterial pathogens associated with BRD risk. The potential for the reduced immunological competence of cattle at feedlot induction can contribute to the severity of acquired infections early in the feeding period. And, finally, the short interval from the feedlot introduction to the peak period of disease risk.

Given the number of pathogens associated with BRD, any mRNA vaccine for this disease will need be polyvalent. It will be essential to determine how polyvalency can be best achieved. As discussed previously for LSDV, one way this could be implemented is by the design and construction of monocistronic mRNA transcripts. It should be equally feasible to develop a multivalent mRNA vaccine through the pooling of pathogen-specific mRNAs during the formulation processes. This approach may be more suited to a disease such as BRD that involves multiple pathogens. As a result of this pathogen diversity, it may be necessary to optimize the expression of antigens in a pathogen-specific manner. Similarly, with appropriate monitoring systems, it may be feasible to formulate vaccines in an informed manner, similar to the use of autologous vaccines in some livestock industries. Underpinning the application of vaccines in this manner could be the emergence of next-generation sequencing technologies to rapidly inform the most appropriate pathogen repertoire for the feedlot of interest [166,167,168]. When combined with other types of genetic testing, pathogen detection via these emerging technologies has the potential to inform vaccine coverage in a highly cost-effective manner [169].

A critical aspect of improving BRD control through vaccination is the need to elicit immune responses quickly. Cattle are at greatest risk of developing BRD during the early feeding period following feedlot induction. Clearly, a conventional vaccination strategy that requires multiple doses within three to four weeks is unlikely to be effective in this scenario. Many of the viral vaccines targeting BRD are MLVs delivered intranasally. The key advantage of this strategy is that the MLV can induce strong innate immune responses in the upper respiratory tract, thus creating an antiviral environment that may provide a broader range of protection than the specific agent or agents included in the vaccine formulation. It remains to be determined if mRNA vaccines can elicit this type of rapid protection as, to date, the major delivery route has been intramuscular injection.

Baldeon Vaca et al. [170] recently reported the potential for LNPs to deliver an mRNA vaccine intranasally in a Syrian golden hamster model for SARS-CoV-2 [170]. The study compared the capacity of two types of LNPs to intramuscular immunization, with two doses delivered three weeks apart for each. While the study reported strong immune responses and protective effects following the intranasal dose installation, these protective effects were not superior to those elicited by intramuscular immunization [170]. These lower immune responses and reduced protections were despite the 5- to 62.5-fold higher mRNA doses delivered to the intranasal-immunized groups compared to the intramuscular groups [170]. While these data suggest that intranasal mRNA vaccination is feasible, further innovations are required to generate equivalent responses to those afforded by conventional immunization routes such as intramuscular.

It possible that the optimal mRNA vaccine for BRD will utilize carrier technologies that prove unsuitable for other applications. As discussed previously, the future development of LNPs is likely to focus on approaches that result in optimal delivery to the early/recycling endosomes. A likely outcome of such improvements is reduced toxicity, resulting in the dampening of innate immune responses in favour of improving adaptive immune responses. However, the BRD-specific challenge of providing cattle with protective immune responses within the most at-risk period for this disease may require a delivery system that stimulates stronger innate immune responses compared to what might be required for other diseases. The challenge will be to achieve this in a controlled manner, so the elicited response does not progress to an inflammatory type of response. Another factor that may permit the use of a more immunostimulatory mRNA vaccine delivery system targeting the BRD-associated pathogens is the reduced risk of disease over time. As discussed above, the BRD risk of cattle diminishes over time as they become acclimatized to the new production environment, therefore reducing the benefits of administering secondary or booster vaccine doses. Indeed, it is generally accepted that management interventions after the period of acclimatization can be detrimental to animal health, and therefore should be avoided. Because of these factors, an mRNA vaccine delivered using a delivery platform that has proven to be unsuitable in multidose vaccine regimes due to the overstimulation of the immune system, particularly in response to secondary doses, could be ideal for targeting BRD-associated pathogens.

One of the key challenges in vaccinating to prevent BRD is the short timeframe that cattle are most susceptible to this disease. Yan et al. [171] recently described the use of an antigen-free immunogen formulation that was able to protect mice against challenge from several nosocomial pathogens. The formulation of aluminium hydroxide, monophosphoryl lipid A, and fungal mannan, delivered subcutaneously, elicited rapid (24 h) and sustained (28 days) innate immune responses that protected mice from bacterial and fungal challenge [171]. Alexander et al. [172] reported that a similar innate immune system stimulant, Amplimune^®^, is safe for use in young beef cattle. Amplimune^®^ consists of the cell wall fractions of *Mycobacterium phlei* formulated with squalene. It has been shown to reduce the clinical signs associated with the enterotoxigenic *E. coli* infection of dairy calves [173]. The coadministration of these innate immune focus vaccines has not been reported to date. Given the specific requirements for improved BRD control through vaccination, the combination of an innate immune stimulation strategy with an antigen-encoding mRNA vaccine is worth testing to determine if the simultaneous eliciting of innate and adaptive immune responses using different immunogens is beneficial to the host.

### 5.6. Barber’s Pole Worm

Helminths, or parasitic worms, are important limiters of livestock productivity [174]. The barber’s pole worm (*Haemonchus contortus*) is the most important internal parasite affecting sheep and goat production globally, particularly in tropical and subtropical regions [175]. Currently, the control of this parasite is mostly with anthelminthics drugs. Where possible, these drugs are used as components of integrated parasite management systems, which can include pasture management, parasite monitoring, nutrition supplementation, and genetic selection. The continued use of anthelminthic compounds has led to the emergence of high levels of resistance in *H. contortus* and other parasitic worms [176]. When combined with other issues, such as environmental persistence and product withholding regulations, the need for alternatives to these compounds is clearly apparent. These drivers have resulted in the investigation of various strategies targeting the development of an effective vaccine for *H. contortus*. Adduci et al. [175] have recently reviewed the immunization strategies that have been evaluated in the search for an efficacious vaccine for *H. contortus*. These strategies include the use of native antigens purified from adults or stage III larvae, recombinant antigens produced using bacterial, insect, or eukaryotic expression systems, and antigens encoded by DNA vaccines [175]. Of those evaluated to date, native antigens and somatic (the polysaccharide component of membrane lipopolysaccharides) antigens purified from adult worms have proven to be the most protective in challenge studies. These antigens can be described as “hidden antigens”, as they are located within the intestinal tissues of the parasite and are therefore “hidden” to the immune system. Host immune responses to these antigens are thought to disrupt parasite feeding and gut integrity, leading to death or reduced fitness [177]. The identification of this class of antigens for *H. contortus* followed the identification of the hidden antigen Bm86, which was effective in eliciting immune responses in cattle and provided protection from infestation from the cattle tick (*Rhipicephalus australis*) following larval challenge [178,179,180].

Two of the most effective *H. contortus* antigens identified to date are native forms of H11 and the H-gal-GP complexes. The variable level of protection afforded by recombinant forms of these antigens have been attributed to several factors, including incorrect folding, the lack of or different post-translational modifications, or potentially undefined components of these preparations. To investigate if inappropriate post-translational modification via the addition of glycans was involved in the reduced efficacy of recombinant H11, Roberts et al. [181] utilized a novel expression system based on the free-living nematode *Caenorhabditis elegans*. Using various approaches, the study confirmed the expression of four known H11 isoforms and one novel isoform that was only expressed by female worms. The study subsequently produced recombinant versions of the five H11 isoforms as soluble proteins using the *C. elegans* expression system. Analysis of the recombinant H11-4 isoform confirmed similar N-linked glycan structures, including highly fucosylated core structures previously reported for the native H11 [181,182]. When sheep were immunized with the coexpressed isoforms H11-4 and H11-5, no differences were detected in adult worm burdens or faecal egg counts compared to the control sheep treated with the adjuvant only. In contrast, the sheep immunized with native H11 had significant impacts on the worm burden and faecal egg counts, with 93.6% and 99.9% reductions, respectively, compared to the controls [181]. Analyses of the serum antibodies detected strong and persistent IgG responses after the second dose, with some IgA and IgM responses detected prior to the second dose for the sheep immunized with native H11. Only IgG antibodies were detected in the sheep immunized with recombinant H11-4 and H11-5 isoforms [181]. The removal of the glycan moieties from either the native or recombinant H11 resulted in significant reductions in antibody binding, confirming the importance of these structures in the epitopes of this antigen [181]. This raises the question of the lack of efficacy afforded by the recombinant isoforms of H11 testing in this study. The authors suggested that a possible explanation was the presence of copurified components of H11 preparations identified by mass spectrometry (referring to a submitted manuscript that does not appear to have been published) [181].

More recently, the complexity of the protein composition and post-translational modifications thereof within the native H11 was confirmed. Wang et al. [183] characterized the glycoproteome of native H11, identifying 85 distinct proteins with 125 N-glycosylation sites. Focusing on the glycan moieties, the authors evaluated the protective efficacies of the formulations containing native H11, denatured H11, or native H11 following the chemical removal of the glycan moieties. Goats immunized with three doses of native H11 or denatured H11 were afforded protection from worm challenge, with significantly reduced adult worm burdens and faecal egg counts. In contrast, goats immunized with the formulation lacking the glycan moieties were afforded minimal protection [183]. Similarly to the previously described study, the protection was mediated by IgG responses, with the highest IgM responses detected for the native H11, followed by decaying responses for the remainder of the experiment [183], while no IgA responses were detected in any of the treatment groups [183]. The study further demonstrated the importance of the glycan moieties by passively immunizing naïve goats with sera from goats with high levels of protection, where significant protection from challenge was confirmed. In contrast, naïve goats passively immunized with sera from goats immunized with the H11 following the removal of glycans were not protected [183].

While efforts continue to define the components of the H11 and H-gal-GP antigen complexes, a formulation containing the native forms has been commercialized in Australia as Barbavax^®^. This vaccine could be described as the most unlikely of veterinary vaccines for the following reasons. Due to the lower efficacy afforded by the same antigens produced in recombinant expression systems, the Barbavax^®^ antigens are purified from adult *H. contortus* that are grown in and recovered from sheep [184]. Following purification from the worms, the antigens are formulated with the adjuvant QulilA [184]. As with the cattle tick antigen Bm86, the *H. contortus* hidden antigen-based vaccination schedule requires multiple doses to elicit immune responses and frequent booster doses to maintain the maximum level of protection. The commercial supplier of Barbavax^®^ provides an online tool to generate the optimal immunization schedule for the successful use of the vaccine [185]. After the first dose, the immunization schedule recommends additional doses at weeks 3 and 7. Further booster doses are recommended at weeks 13, 19, and 26. The administration of a final dose at week 25 is also suggested, depending on the weather conditions at that time. The manufacturer recommends that the vaccine be used as part of an integrated worm management plan; thus, the continued monitoring of faecal egg counts is strongly recommended from 11 to 12 weeks postimmunization, and augmenting vaccine-based control with an appropriate anthelmintic if high worm burdens are detected. The successful commercialization of this vaccine demonstrates that, where there is sufficient need, in this case driven by production losses and the emergence of resistance, many perceived impediments to vaccine adoption by livestock industries can be overcome. While Barbavax^®^ is clearly a vaccine success story, the current manufacturing processes and recommended immunization schedule make the delivery of this vaccine to the at-risk livestock populations in low- and middle-income countries a difficult proposition. It is the livestock populations of these countries where an *H. contortus* vaccine would have the most impact, making a major contribution to the achievement of the relevant SDGs.

Further research is required to determine how the hidden antigens known to provide high levels of efficacy against *H. contortus* can be utilized in the development of an mRNA vaccine. One issue that is common between the hidden antigen-based vaccination against *H. contortus* and cattle ticks is the requirement for multiple doses to elicit protective immune responses, followed by frequent booster doses to maintain protection. As these antigens are components of the intestinal tissues of these parasites, despite constant exposure to the parasites, there is no natural re-exposure of these antigens to the host immune system, and therefore no natural boosting of the immune responses. Thus, strategies that aim to increase the duration of the immunological responses to these antigens warrant further investigation. Approaches such as the use of molecular-based adjuvants fused to the hidden antigens could be amenable to mRNA-based delivery strategies to improve the duration of the immune responses observed following the administration of the primary doses. The C3d component of the complement system and its derivatives have been evaluated as molecular adjuvants to enhance immune responses, particularly antibody responses, to antigens from a wide variety of pathogens, including parasites. The applications and strategies used for C3d and other potential molecular adjuvants are reviewed by Sicard et al. [186]. Briefly, this approach has typically involved the fusion of the antigen and molecular adjuvant coding sequences; as such, molecular adjuvants could be readily incorporated into a prototype mRNA vaccine during the design phase. The use of a molecular adjuvant is likely to be application-dependent due to the tendency for a strong bias towards Th2-dominant immune responses [186].

If the antigenic components of Barbavax^®^ are to be used for the development of an mRNA vaccine for *H. contortus*, the components of these antigen complexes and their specific roles in protection will need to be more clearly defined. The recent report that native H11 contains 85 proteins suggests that novel approaches are likely to be required to achieve this. It may well be that mRNA vaccination will play a role in fulfilling this need. The demonstration that the glycan moieties of native H11 are key determinants of immunological protection suggest that an mRNA vaccine based on some components, if not all, of this antigen may not be feasible due to the key differences in the post-translational glycan structures added to the proteins by the parasite compared to those added by the host. However, combining the previously described *C. elegans* expression system with mRNA technology may enable the development of a more readily adoptable vaccine. Combinatorial mRNA libraries encoding of the individual components of the H11 complex could be constructed and used to produce each antigen for evaluation using in vitro assays and/or in vivo testing. Similarly, this approach could be used more broadly to identify new protective antigens of *H. contortus* and potentially other parasites. It is plausible that this approach may identify additional protective antigens where parasite specific post-translational moieties are not the key determinants of protection, thus providing a potential pathway to an mRNA vaccine for *H. contortus*.

## 6. Summary

Many diseases examined in this review could be considered neglected, emerging, or re-emerging diseases of livestock, and therefore they have continued to limit the productivity and economic development in affected countries. However, in recent years, some of these diseases have continued to spread from what might be considered their native distributions. This is best exemplified by the recent emergence of the LSDV and PPRV in areas of Southeast Asia. While the underling drivers and mechanisms this phenomenon are complex, the need for safe and effective vaccines has become more apparent.

Clearly, the accelerated development of mRNA vaccines and associated technologies driven by the COVID-19 pandemic have rapidly matured this vaccination platform that has the potential to address infectious diseases of global importance in animal production. What is equally evident is that, while improved vaccines will help to achieve the SDGs, they will not be the silver bullet that some might think they will be.

While the rapid development and deployment of mRNA vaccines has been questioned by some sections of the global community, this debate has typically not encompassed the complete history of this technology platform. What may have appeared to be the sudden appearance of a “new vaccine technology” was anything but. The sudden emergence of the COVID-19 pandemic and its impact on human health resulted in an unprecedented focus on vaccine technologies. The response of governments was to remove the major impediment to vaccine development, namely, investment. The government of the United States of America alone invested USD 17B through *Operation Warp Speed* to accelerate the development of COVID-19 vaccines [15]. This level of investment removed the major hurdle of financial risk faced by the pharmaceutical industry of securing the funding required to accelerate laboratory studies and to commence the required clinical trials. Prior to the pandemic, the estimates for taking a human vaccine from conceptualization through to phase IIa clinical trials (efficacy studies) was estimated to range from USD 360 to USD 500M [187,188]. Consequently, the decision to undertake the development of a vaccine is not taken lightly.

Pronker et al. [189] estimated that it takes on average 10.7 years to move a prototype vaccine from the preclinical developmental phase through to registration, with only 6% of vaccines successfully negotiating this developmental pipeline. More recently, Gouglas et al. [187] utilized a stochastic modelling approach to evaluate the potential costs associated with progressing the development of human vaccines for 11 diseases of pandemic potential. Considering the number of candidate vaccines and their respective stages of development for the 11 diseases of interest, it was estimated that the cost of developing a single vaccine for one of these diseases to the end the of phase 2a trials would be USD 31–68 M (USD 14–159 M range), if success is assumed. Including the risk of failure in the modelling considerably increased the associated costs. Overall, the estimated cost of successfully developing at least one vaccine for each of the diseases of interest was estimated at USD 2.8–3.7 B (USD 1.2–8.4 B range) [187]. Clearly, the decision to commit to the development of a human vaccine in a commercial setting is not undertaken lightly. It is reasonable to conclude that the cost of developing a veterinary vaccine is likely to be considerably less, particularly with the equivalent developmental stages to phase I and phase IIa trials being more cost-effective in animal species. However, these figures are provided to illustrate the impact of the investment in human vaccine development resulting from the pandemic and how it compressed the vaccine developmental pipelines, particularly with a no-financial-risk scenario created by the massive public investment [190].

Prior to the COVID-19 pandemic, it was estimated that the cost of the GMP production of mRNA vaccines using IVT could be up to ten-fold lower compared to the GMP production of proteins for therapeutic use [191]. Though, as discussed previously, this review of the cost–benefit ratio for mRNA vaccines over subunit vaccines is likely to be less (Table 1). The substantial investment in mRNA production and vaccination systems in response to the pandemic is likely to have, or have led to, further improvements in the cost–benefit ratio. Such a highly beneficial cost of production ratio, combined with the flexibility of mRNA production, clearly suggests a very important role in reducing the losses of productivity in ruminant-based protein production, thus making an important contribution to the achievement of the relevant SDGs.

## 7. Conclusions

As demonstrated by Susuki et al. [88], altering the LNP composition alters the in vitro and in vivo properties of the LNP–mRNA formulations. This capacity could enable the development of LNP–mRNA vaccines to address key challenges in livestock vaccine adoption. Firstly, the capacity to increase the stability in mRNA formulations for longer periods at higher temperatures following lyophilization would facilitate the adoption of this technology for livestock applications. In subtropical and tropical production systems, the need for cold-storage chains to maintain vaccine potency can be difficult, if not impossible, for those in countries with developing economies, where the SDGs are particularly important. The storage requirements for leading COVID-19 mRNA formulations ranged from −20 °C to −80 °C for a six-month shelf-life when lyophilized and when the reconstituted potency was maintained for <30 days with refrigeration [192]. Thus, improving the shelf-life through the optimization of the LNP composition is likely to enhance the suitability of the mRNA vaccination of ruminants and other livestock. Secondly, the capacity to optimize the persistence of mRNA formulations in a specific subset of tissues in food-producing animals, whilst not compromising the generated immune response, is likely to be a very important attribute. Being able to predict and confirm this attribute will be important to underpin regulator and consumer acceptance alike. The recent development of LNPs that can specifically target muscles clearly demonstrates the feasibility of this type of approach [91].

The route of vaccination is also an important consideration in the application of veterinary vaccines. Injectable vaccines are widely used in veterinary medicine, and whilst largely successful, they can be problematic with respect to operator safety and injection-site reactions. There is also the question of whether delivery via injection can induce effective immune responses at mucosal surfaces, which are the first point of contact between the host and pathogens of the respiratory, reproductive, and digestive systems. Thus, the potential innovations for LNP–mRNA delivery to mucosal surfaces could also improve the adoption of this technology by livestock producers.

It is clear that, for some of the example ruminant diseases discussed here, there is the potential for the direct translation to the use of mRNA vaccine technologies to address the deficiencies in current vaccination strategies. Importantly, mRNA vaccination offers considerable advantages over the current technologies that are widely used in veterinary medicine, including the capacity to be readily modified to match any changes in the circulating strains of the pathogens of interest and to induce balanced immune responses with no risk of reversion to virulence, in contrast to subunit vaccines, inactivate vaccines, and MLVs.

However, for some diseases of interest, specific challenges must be met to enable the utilization of mRNA vaccinations to improve disease control and reduce productivity losses. For largely neglected diseases, such as LSD, the identification of protective antigens remains a critical knowledge gap, while for BRD, the key challenges are the efficient incorporation of multivalency and the reduction in the time to immune potentiation to protect animals when they are most at risk. For the vaccines used in applications such eradication campaigns or the control of exotic disease outbreaks, the capacity to utilize robust DIVA principles is essential. The innate flexibility of mRNA vaccine design and production suggests that the technology is ideally suited to solving these ruminant health challenges and to the delivery of sustainable improvements in productivity.

A recent report prepared by Oxford Analytica for HealthforAnimal has estimated that, globally, USD 982 M is lost daily because of productivity losses in livestock industries [193]. The report also estimated that every percentage point reduction in global beef cattle losses due to disease could provide sufficient products to meet the needs of 317 M people (addressing SDG-1 to 3), while a 10% reduction in disease levels could reduce greenhouse gas emissions equivalent to 800 M tonnes of carbon dioxide annually (addressing SDG-12 to 15). Clearly, safe and efficacious vaccines have the capacity to deliver these outcomes, and therefore contribute to achieving these SDGs. As mRNA vaccination technologies continue to develop and are applied to ruminant diseases, they will play important roles in delivering these outcomes. The major challenge that remains is for the relevant stakeholders, such as governments, peak industry bodies, and veterinary health companies, to provide the investment required ensure mRNA vaccines are developed for ruminants and other livestock to enable these productivity gains to be realized.

In conclusion, the continuing advances in mRNA vaccine technologies suggest this rapidly evolving platform will have the capacity to improve the control of ruminant diseases that have proven difficult to control with conventional vaccination strategies. Advantages such as the low cost of production, the capacity for multivalency and flexible formulation, and the potential for the elimination of cold chains for storage and transport suggest that mRNA vaccines will become an integral component in improving the efficiencies in livestock production required to achieve the relevant SDGs.

## Figures and Tables

**Table 1:** Comparison of the estimated cost of manufacturing selected vaccine technologies on a per-dose basis. The costs shown are for messenger RNA (mRNA), self-amplifying RNA (saRNA), attenuated bacterial (attBac), modified live virus (MLV), recombinant subunit (Rec. subunit), and adenovirus-vectored (AdV vect.) vaccines. Cost estimates are shown in US dollars.

	Dose Characteristics				
Vaccine Type ^1^	Quantity	Scale ^2^	Cost	Comment	Reference
mRNA	100µg	N.A. ^3^	USD 2.85	Modern ^4^	[28]
mRNA	30 µg	N.A. ^3^	USD 1.18	Pfizer ^4^	[28]
mRNA	100 µg	345 M	USD 2.02	30 L	[26]
mRNA	30 µg	1.15 B	USD 0.61	30 L	[26]
mRNA	12 µg	2.876 B	USD 0.20	30 L	[26]
saRNA	1 µg	8.554 B	USD 0.02	30 L	[26]
saRNA	0.1 µg	12.22 B	USD 0.0043	30 L	[26]
attBac	N.S.	20 M	USD 2.62 ^5^	One vaccine	[27]
MLV	N.S.	20 M	USD 2.36 ^5^	One vaccine	[27]
Rec. Subunit	N.S.	20 M	USD 2.68 ^5^	One vaccine	[27]
attBac	N.S.	100 M	USD 2.12 ^5^	Five vaccines ^6^	[27]
MLV	N.S.	100 M	USD 1.95 ^5^	Five vaccines ^6^	[27]
Rec. Subunit	N.S.	100 M	USD 1.84 ^5^	Five vaccines ^6^	[27]
AdV vect.	N.S.	400 M	USD 0.15	Batch culture	[29]
AdV vect.	N.S.	400 M	USD 0.23	Perfusion culture	[29]

^1^ Vaccines formulated and ready for use. ^2^. Number of doses manufactured in millions (M) or billions (B). ^3^ Not applicable. ^4^ Manufacturer. ^5^ Costs are based on vaccine manufacturing in a developing country. ^6^ Reduced costs of manufacturing due to economies of scale and the scope of producing five compared to one vaccine within the same infrastructure.

## Data Availability

No new data were generated in the preparation of this manuscript.
